# Yeast Mating and Image-Based Quantification of Spatial Pattern Formation

**DOI:** 10.1371/journal.pcbi.1003690

**Published:** 2014-06-26

**Authors:** Christian Diener, Gabriele Schreiber, Wolfgang Giese, Gabriel del Rio, Andreas Schröder, Edda Klipp

**Affiliations:** 1 Theoretische Biophysik, Humboldt-Universität zu Berlin, Berlin, Germany; 2 Instituto de Fisiología Celular, Universidad Nacional Autónoma de México, Circuito Exterior S/N Ciudad Universitaria, México D.F, México; 3 Institut für Mathematik, Humboldt-Universität zu Berlin, Berlin, Germany; Chalmers University of Technology, Sweden

## Abstract

Communication between cells is a ubiquitous feature of cell populations and is frequently realized by secretion and detection of signaling molecules. Direct visualization of the resulting complex gradients between secreting and receiving cells is often impossible due to the small size of diffusing molecules and because such visualization requires experimental perturbations such as attachment of fluorescent markers, which can change diffusion properties. We designed a method to estimate such extracellular concentration profiles *in vivo* by using spatiotemporal mathematical models derived from microscopic analysis. This method is applied to populations of thousands of haploid yeast cells during mating in order to quantify the extracellular distributions of the pheromone **α**-factor and the activity of the aspartyl protease Bar1. We demonstrate that Bar1 limits the range of the extracellular pheromone signal and is critical in establishing **α**-factor concentration gradients, which is crucial for effective mating. Moreover, haploid populations of wild type yeast cells, but not *BAR1* deletion strains, create a pheromone pattern in which cells differentially grow and mate, with low pheromone regions where cells continue to bud and regions with higher pheromone levels and gradients where cells conjugate to form diploids. However, this effect seems to be exclusive to high-density cultures. Our results show a new role of Bar1 protease regulating the pheromone distribution within larger populations and not only locally inside an ascus or among few cells. As a consequence, wild type populations have not only higher mating efficiency, but also higher growth rates than mixed *MAT* **a** *bar1Δ/MAT* **α** cultures. We provide an explanation of how a rapidly diffusing molecule can be exploited by cells to provide spatial information that divides the population into different transcriptional programs and phenotypes.

## Author Summary

Haploid budding yeast cells cannot actively move to find a mating partner, like some flagellated bacteria do. Instead they must grow a so-called shmoo – a mating projection – precisely into the direction of a potential partner. They communicate with each other by releasing pheromones into their environment, which are sensed by cells of the opposite mating type. This serves the localization of nearby cells and initiates growth arrest and mating. Paradoxically, yeast cells also secrete the protease Bar1 that destroys pheromones. To visualize the resulting pheromone distribution and understand the effect on mating efficiency, we combined fluorescence imaging and mathematical modeling. We observed that the controlled destruction of pheromones by the yeast cells is beneficial to communication since it causes relatively higher pheromone concentrations in areas where cells are dense and vanishing pheromone concentrations elsewhere. This allows the population to maintain two different cellular behaviors at the same time, i.e. mating and continued growth, a behavior which disappears when we genetically delete the gene for the pheromone-destroying protein.

## Introduction

Cells communicate with each other by detecting and responding to external cues and stimuli. In cellular systems one can find several examples where cells coordinate growth in specific regions by sensing and responding to small differences in the concentrations of substances that provide positional information [1], [2]. Such signaling among cells is a common feature of cell populations as well as multicellular organisms, where cells often operate close to the physical limit of gradient or concentration detection [3]–[5].

The pheromone response of budding yeast (*Saccharomyces cerevisiae*) is an example for a eukaryotic cell communication system. Yeast cells occur either in the haploid forms *MAT* **a** and *MAT* **α**, or as *MAT* **a/α** diploid. Haploid and diploid cells are both able to replicate vegetatively. Mating of two haploid cells with opposite mating types yields diploid cells, while haploid cells are formed through spore formation in meiosis [6], [7].

Mating is initiated by the secretion of mating type-specific pheromones, called **a**-factor and **α**-factor, which are sensed by haploid cells of the opposite mating type and trigger the mating response [8], [9]. During the mating response, yeast cells arrest their cell cycle in G1 phase and elongate in the direction of the pheromone signal by forming directed mating projections called “shmoos” [6], [10]. Yeast can sense pheromone gradients as well as absolute concentration levels. Nevertheless, yeast cells are not capable of chemotaxis and, thus, mating requires the haploid cell types to signal their location particularly to nearby potential mating partners. One way for *MAT* **a** cells to regulate the extracellular **α**-factor is the secretion of Bar1, an aspartyl protease, which degrades **α**-factor [11], [12]. This leads to the paradox that *MAT* **a** cells degrade the signal they need to receive. Theoretical investigations hypothesized that the major role of Bar1 is to sharpen the pheromone gradient [13], [14].

However, direct visualization of the resulting spatial **α**-factor concentration profiles between secreting and receiving cells is impossible due to the small size of diffusing molecules and because such visualization requires experimental perturbations such as attachment of fluorescent markers, which change diffusion properties and activities. Therefore, reaction-diffusion (RD) models have been used to simulate the pheromone distribution on the basis of physical properties of the molecules [13]–[16], but neither the validity of the model predictions nor the effect of the pheromone distribution have been tested experimentally. Except of the publication of Jin *et al.* [15], where it has been suggested that Bar1 promotes avoidance of the same mating type and accurate gradient detection. Using a microfluidic device it had been shown how *MAT* **a** cells avoid each other when exposed to an artificial unidirectional gradient, which was reproduced quite vividly by simulations of an RD model. However, the assumptions and choice of parameters were in contrast to other works [14], [16] (compare Text S1). In general, experimental validation of RD models is complicated, especially on the molecular level [17].

Moreover, recent theoretical findings support the theory, that secretion of Bar1 in the extracellular medium does not help to align gradients in the direction of the opposing mating type [16]. In summary, the role of Bar1 is still controversially discussed. Also, none of the models proposed so far has investigated interactions of more than a few cells or the four haploid spores in an ascus, even though mating occurs not only inside the ascus, but also in a cell population which was shown by new findings where a remarkably high outcrossing rate from asci was reported [18]. This indicates that mating yeast cells interact with quite a number of potential mating partners in a natural environment. Furthermore, a recent study has shown the potential of simple secrete and sense motifs to exhibit surprising effects on the population level [19].

Therefore, we designed a method to identify the most likely **α**-factor distribution within mixed haploid yeast populations of thousands of cells directly from confocal microscopic images with fluorescently tagged marker proteins. Here, an RD model is used to simulate interactions of a few hundred cells at the same time. In *MAT* **a** cells, the protein Fus1, which is strongly expressed upon pheromone stimulation, is tagged with GFP to record pheromone pathway activation and serves as a proxy for Bar1 induction. Therefore, the experimentally observed pheromone activation level of each *MAT* **a** cell is integrated into the model and compared to the experiments. *MAT* **α** cells are modified to express mCherry from the TDH3 promoter to indicate their mating type and location. In a unique way we coupled physical RD models with experimental imaging in order to quantify the spatial distribution of extracellular **α**-factor. The use of simple marker constructs, that altered neither **α**-factor nor Bar1, served for minimal interference with the biological system. We used this approach to directly estimate the influence of Bar1 on the distribution of **α**-factor in a mixed yeast population and suggest a novel function of Bar1 to enable the coordination of mating and growth in a yeast population *in vivo*.

## Results

### Extracellular α-factor concentrations were quantified along with Bar1 activity directly from *in vivo* confocal images

**Figure 1:**
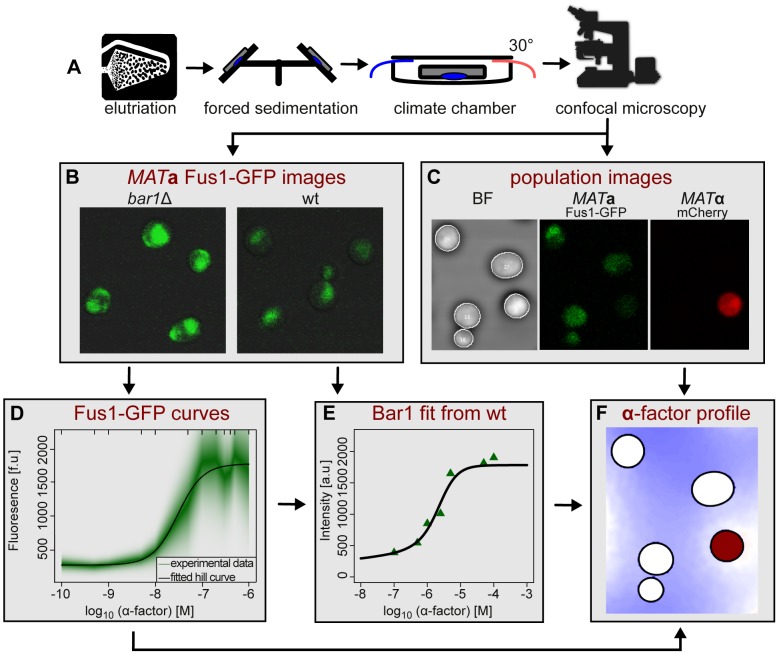
The combined experimental and computational methodology used to derive the extracellular distribution of α-factor. (A) Cells were synchronized by elutriation, fixed by forced sedimentation, and microscopically analyzed. (B) Fus1-GFP was measured in *MAT* **a** wild type and *bar1Δ*cells after stimulation with various levels of **α**-factor (displayed here: **α**-factor concentration of 500 nM) and used for calibration curves in (D) and (E). (C) Overlays of the bright field, mCherry and GFP channels allowed recording the positions of *MAT* **α** (in red) and *MAT* **a** cells for further mathematical analysis. (D) Calibration of the response of *bar1Δ* cells to given amounts of **α**-factor allowed to estimate, how much **α**-factor would reach the cell without degradation by Bar1. (E) Calibration of the response of wild type *MAT* **a** cells enabled calculation of Bar1 secretion in response to **α**-factor. (F) The information obtained in panels (B) to (E) was used to calculate the unknown distributions of Bar1 and **α**-factor in the space between the cells (**α**-factor distribution is displayed).

We combined image analysis with spatiotemporal mathematical modeling to determine spatial concentration distributions of Bar1 and of **α**-factor. Figure 1 introduces the concept of the approach:

- Take images, detect cell location and mating type, and quantify pheromone stimulation of *MAT* **a** cells with a fluorescent marker.
- Use a mathematical model based on real cell location and activation to calculate the distribution of Bar1 and **α**-factor in the extracellular space.
- Predict effects on population growth and mating efficiency, confirmed by further experiments.

In order to obtain *in vivo* conditions during microscopy, which were suited for the described methodology, confocal microscopic images were taken from synchronized haploid cells or from equally mixed haploid *MAT* **a**/*MAT* **α** cell populations. The cell culture samples were spun down with low g-force on glass bottom dishes in order to have immobile cells without using concanavalin A coated dishes (which we found to alter the population response). This protocol essentially yielded sedimented cells in the same way as they would be present in any laboratory or naturally occurring non-agitated medium, with the difference that sedimentation here was achieved under controlled conditions.

Location, shape, and mating type of the cells were extracted from out-of-focus images in the brightfield and mCherry channel (Figure S1 in Text S1). The cells' spatial arrangement on the images was transferred into locally refined triangular meshes for the model (Figure S2 in Text S1) and used to calculate the extracellular spatial distributions of Bar1 and **α**-factor.

In mathematical terms the problem (ii) is described as a pure extracellular reaction-diffusion process for **α**-factor and Bar1 with distinct boundary conditions (Figure S3 in Text S1). We formulated an RD model by the following set of partial differential equations:
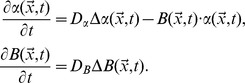
This system covers the extracellular dynamics of **α**-factor concentration, 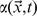, and the activity of Bar1 protease, 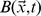, over time *t* and position in space, 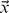. The equations quantify two types of processes: (1) diffusion of both *α* and *B*, where 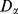 and 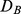 are diffusion constants and Δ denotes the Laplacian, and (2) degradation of *α* by *B*. Boundary conditions at the cell surfaces define secretion of **α**-factor by *MAT* **α** cells and induced secretion of Bar1 by *MAT* **a** cells.

The diffusive flux of **α**-factor on the cell surface and exterior system boundaries is given by:
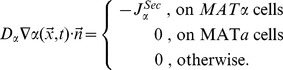
Here, 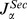 is a constant specifying the secretion of **α**-factor. The vector 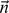 points towards the cell interior and, therefore, the diffusion flux 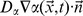 at the boundary takes a negative value for the secretion of molecules and a positive sign for the absorption of molecules. The induction of Bar1-activity at each *MAT* **a** cell is calculated from the average **α**-factor concentration at the surface of this cell, which is represented by a Hill-curve (compare Figure 1 E, for details see Figures S4 and S5 in Text S1):
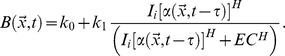
The expression 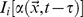 is the average concentration of **α**-factor at the *i*-th *MAT* **a** cell at time 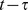, which promotes the secretion of Bar1. We assume a zero Bar1 flux on the *MAT* **α** cells and the system exterior:
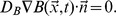
The remaining exercise was to identify the parameter values of the RD model. Diffusion constants for the proteins were directly calculated from protein properties (size and density). Thus, only two parameters had to be identified by parameter estimation: the activity of Bar1 and the secretion rate of **α**-factor, as described below.

To determine the activity of Bar1, we used a three-step procedure. First, we performed an initial calibration to quantify the **α**-factor concentration perceived by individual *MAT* **a** cells. We used *MAT* **a** cells carrying a *BAR1* deletion (*bar1Δ*) and the pheromone response marker Fus1-GFP and synchronized them in G1 phase, where they are responsive to pheromone. Their response to varying concentrations of **α**-factor (artificially added, in the absence of *MAT* **α** cells) was quantified as Fus1-GFP fluorescence intensity on microscopic images. Since *bar1Δ* strains do not secrete the protease, the local **α**-factor concentration was equal to the applied concentration. Fluorescence intensity of Fus1-GFP in correlation with **α**-factor concentration was recorded as a calibration curve (see Figure S4 in Text S1). The calibration curve was then applied to mixed haploid cultures to determine the perceived **α**-factor concentration for each *MAT* **a** cell on the image (see Figure 1). In mathematical terms, the calibration yielded the boundary value of **α**-factor concentration at the surface of a *MAT* **a** cell and a functional relation between this **α**-factor concentration and the induced Bar1 expression.

Second, the steady state activity of Bar1 was quantified by stimulating wild type *MAT* **a** cells carrying the Fus1-GFP marker in G1 phase with given concentrations of **α**-factor. Since there were no *MAT* **α** cells, the **α**-factor secretion rate was equal to zero at this stage. For the model, these data were used in a mathematical optimization to quantify the activity of Bar1 for wild type cells. In order to verify that Fus1 can in fact be used as a proxy for Bar1, we used a strain expressing qVenus fluorophore under the control of the Bar1 promoter (Bar1pr-qVenus, [20]) while maintaining wild type Bar1 activity (see Figure S5 in Text S1 and Materials and Methods). The induction occurred with the same kinetics and Hill-coefficients as the Fus1 induction, but with a slight delay of the Bar1 expression verifying our used induction kinetics for Bar1. The Bar1pr-qVenus construct was able to quantify the expression levels of Bar1, but not the Bar1-induced degradation rate of **α**-factor in the extracellular medium, which is why we preferred the use of the Fus1-GFP data.

**Figure 2:**
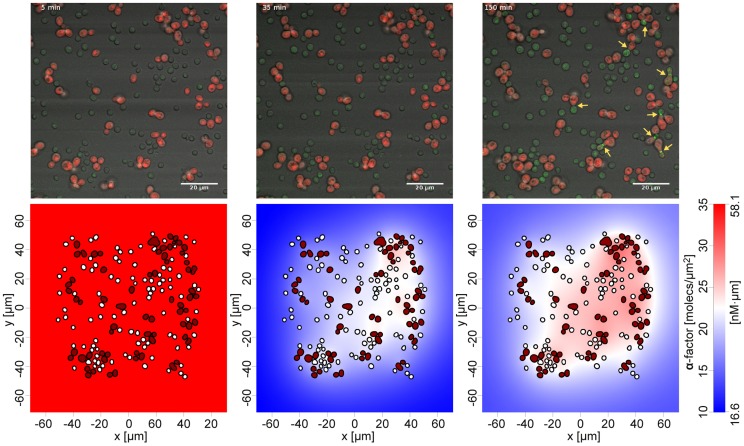
Time course of pheromone distribution. Shown are overlay images of Fus1-GFP and mCherry (2A–2C) and the simulated pheromone distributions (2D–2F) at three distinct time points. Yellow arrows indicate mating cells after 2.5 hours. Note that the occurrence of mating events corresponds well with areas of high pheromone concentrations and gradients. The extracellular **α**-factor distribution is indicated on the color scales bar. Note that **α**-factor is recorded in a 3-dimensional volume and projected on the 2-dimensional plane related to the image plane. This results in units molecules per µm^2^ or, alternatively, nM·µm, as indicated. The full time course is provided in Supporting Movie S1.

**Figure 3:**
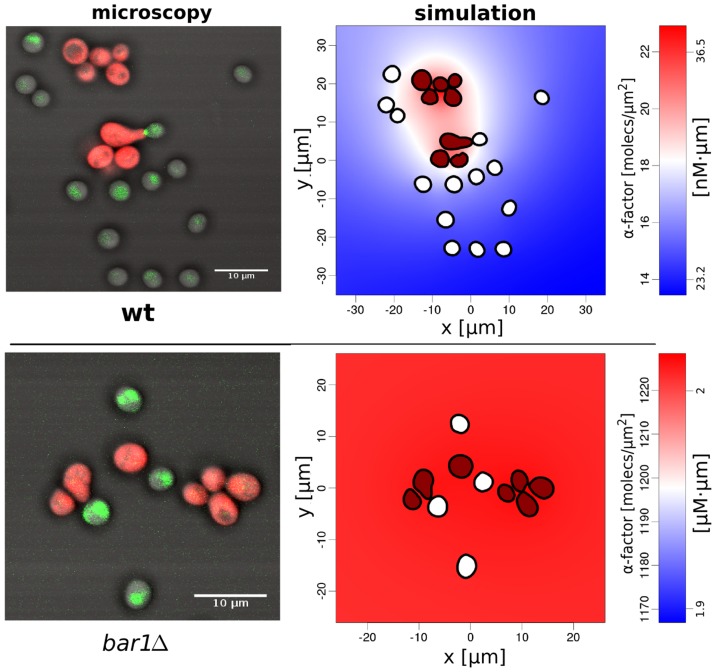
Microscopic images (left) and derived computational domains and α-factor distributions (right) for *BAR1* wild type (top) and *bar1Δ* (bottom). The microscopic images are an overlay of the bright field, mCherry (for *MAT* ***α***) and GFP channels (for Fus1 expression in *MAT* **a**). Individual images are given in Figure S1 in Text S1. Computed images show *MAT* ***α*** in red and *MAT* **a** in white. The extracellular **α**-factor distribution is as indicated on the color scales. Note different scales for **α**-factor for wild type (top) and *bar1Δ* (bottom).

Third, the **α**-factor secretion rate could be calculated by parameter optimization from images of mixed haploid yeast cultures containing *MAT* **a** Fus1-GFP cells and *MAT* **α** mCherry cells and the information we obtained for the Bar1 activity. Due to the lack of evidence for an extracellular protease activity in *MAT* **α** cells, we neglected potential differences in **a**-factor induction of **α**-factor. In practice, the induction of **α**-factor secretion should be nearly homogeneous on a single image, but may vary for different images. We obtained secretion rates from significant fits (F-test p<0.05) of 9 images (see Text S1). The mean fitted value of the **α**-factor secretion rate of 865 molecules per second and *MAT* **α** cell is in good agreement with recent experimental measurements (550 molecules per second and cell measured as basal secretion with 2.5 - 4-fold maximal induction) [21], [22]. The fully parameterized model could now be used to efficiently calculate the entire **α**-factor distribution on arbitrary images. We validated the obtained model and parameters by predicting the Fus1-GFP fluorescence on an image of a larger mixed yeast population not used for model fitting (F-test p<2.2e-6). The power of this combined imaging and modeling approach is illustrated in Figures 2 and 3 and in Movie S1, which shows a mixed haploid population during growth and mating next to the simulation of the distribution of **α**-factor on a computational grid generated directly from the corresponding microscopic image.

### Global Bar1 activity limits the range of the α-factor signal and optimizes its information content

We observed large differences in the estimated local **α**-factor concentrations between wild type cell populations and cell populations with a *bar1Δ* background. Dense wild type cell populations showed a strongly localized **α**-factor distribution at sites of high *MAT* **α** cell density, with **α**-factor concentration quickly declining with distance. Consequently, *MAT* **a** cells far away from a cluster of *MAT* **α** cells experienced significantly lower local **α**-factor concentrations than close-by cells, and hence were often non-permissive for induction of the pheromone response (Figure 3). Populations with *bar1Δ* background showed an almost uniform distribution of very high pheromone concentrations, resulting in global pathway activation as evidenced by high Fus1-GFP expression. Nevertheless, the global (over-) activation led to reduced mating events.

**Figure 4:**
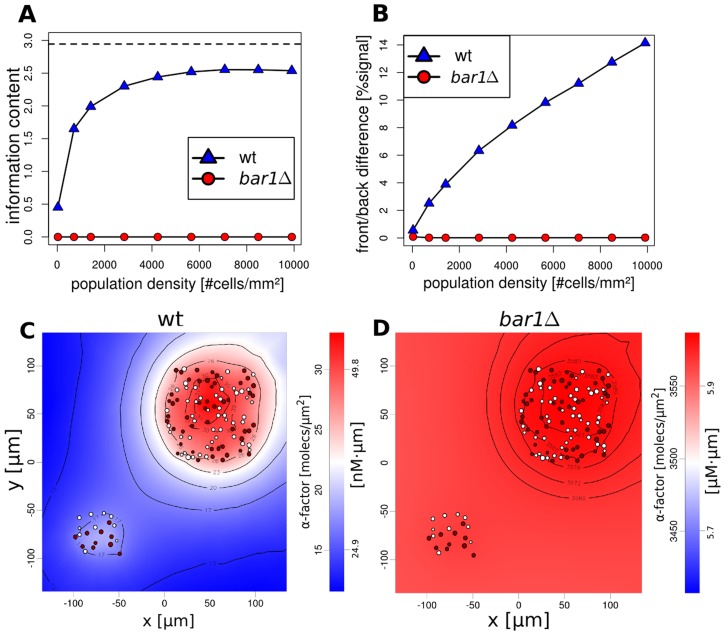
Virtual cell populations were randomly generated in order to track the influence of population density on the α-factor distribution. (A) Maximum information content of the **α**-factor distribution as calculated by entropy (see Text S1) depending on population density. (B) Average **α**-factor gradients (relative front/back difference, see Text S1) for individual cells in the populations. (C and D) Calculated **α**-factor distributions for subpopulations of different densities in wild type and *bar1Δ*.

We wanted to see whether this behavior arises in general and independently of the exact spatial composition of the culture. Thus, we performed a computational study using randomly generated cell populations mimicking the ones observed microscopically with varying cell densities (Figure 4). Each virtual population was simulated both with wild type Bar1 secretion and in *bar1Δ* background. We tracked key parameters such as the average **α**-factor concentration, the pheromone gradients perceived by the individual *MAT* **a** cells (calculated as the average difference in **α**-factor concentration a cell would sense at its shmoo tip and the opposing cell site), and the maximum information content of the **α**-factor distribution. Information (or Shannon entropy) quantifies the “surprisal” of a specific **α**-factor concentration, i.e. how likely an observed **α**-factor concentration is given an overall **α**-factor distribution obtained for many cell populations.

Virtual wild type populations exhibited a strong gain for the information content of the **α**-factor distribution as the population size increases (Figure 4A). This was accompanied by increasing **α**-factor gradients across *MAT* **a** cells (Figure 4B). In contrast, populations not secreting Bar1 showed information contents close to zero (Figure 4A) as well as insignificant pheromone gradients (Figure 4B), both independently of population density. We noted that the overall pheromone concentration remained within a range of up to 20 nM in wild type, but in the mutant linearly increased with population density (see Figure S6 in Text S1). This observation indicates that the gradients and, thus, the reachability of nearby mating partners can only be detected faithfully in cell populations secreting Bar1, particularly in high cell densities.

Additionally, we simulated various scenarios where a high-density subpopulation was placed next to a low-density subpopulation (Figure 4C–D). Here, the wild type is capable of limiting the **α**-factor distribution to the corresponding subpopulation, leaving the low-density subpopulation unaffected by the high local **α**-factor concentration of the high-density subpopulation. The same also holds true for random cell distributions (see Figure S7 in Text S1). Again, this behavior was not observed in the absence of Bar1, showing that Bar1 activity restricts the distribution of **α**-factor. Hence, only subpopulations with high local cell densities and small intercellular distances, as required for successful mating, were exposed to **α**-factor concentrations permissive for mating.

**Figure 5:**
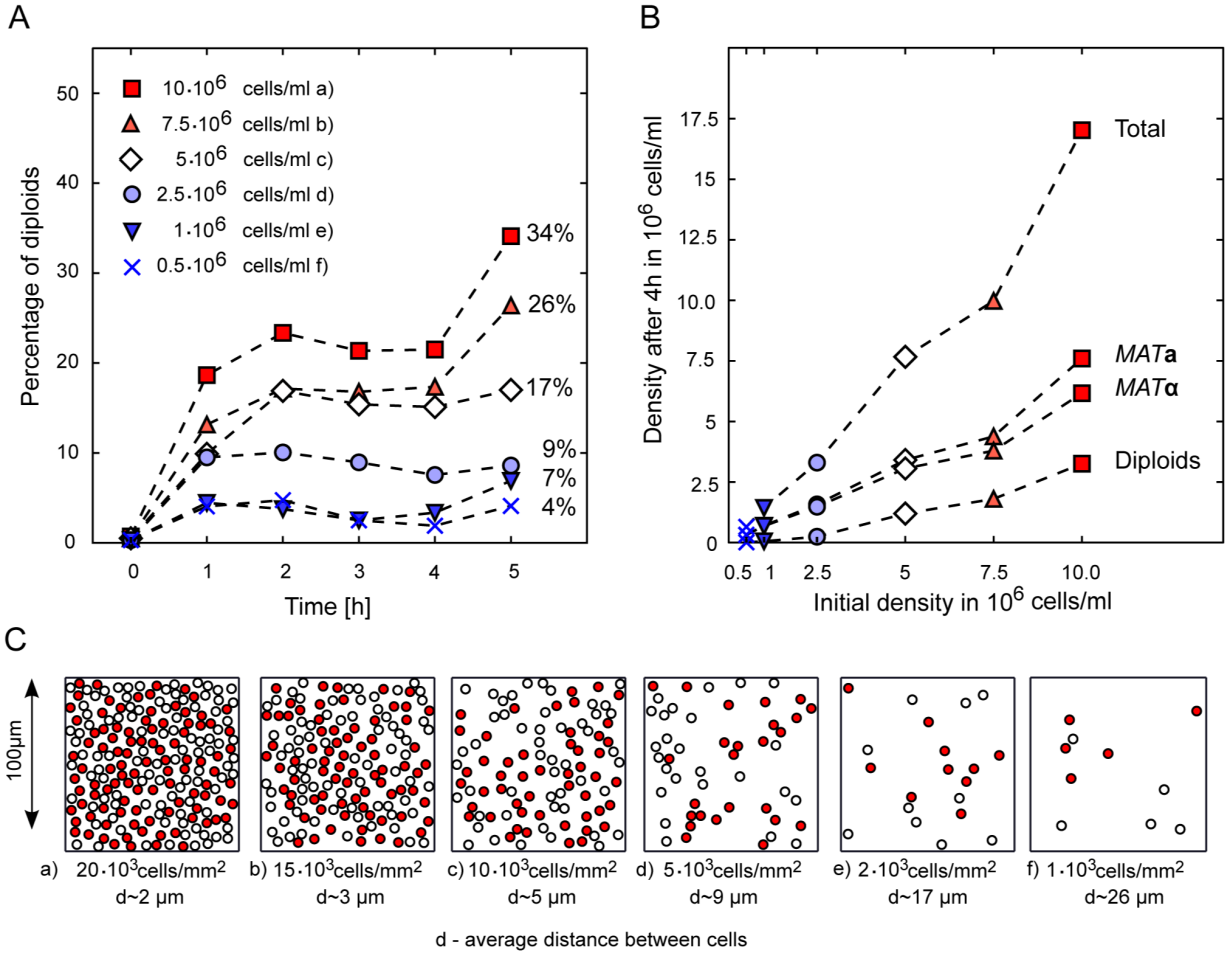
Density dependence of cell growth and diploid formation. (A) Fraction of diploids in mixed populations of different cell densities measured over time (each data point comprises between 5.000 and 260.000 counted cells). Indicated is the starting condition at time 0 h. (B) Absolute cell numbers of *MAT* **a**, *MAT* ***α*** and diploid cells at 4 h. Note the stronger increase in cell numbers of *MAT* **a** compared to *MAT* **α**. (C) Examples of intercellular distances for populations with various cell densities. The shown densities correspond to the ones used in the experiments. Density calculations are provided in Text S1, simulated distributions of **α**-factor and Bar1 activity are given in Figure S7 in Text S1.

We could validate this model prediction concerning the dependency of mating success on culture density experimentally by incubating mixed *MAT* **a** Rpl9A-GFP/*MAT* **α** mCherry populations in cell densities varying from 0.5–10 million cells/ml for various time-points up to 5 h. We incubated the cells in Petri dishes of 36 mm diameter to ensure that the experimental density in the resulting cell layer is in agreement with the simulated density (density and distance calculations can be found in Text S1). Subsequent cell counting during bead-normalized flow cytometry allowed us to quantify the absolute number of each cell type along with the number of diploid cells in each sample (Figure 5). Here we also observed a strong dependency of diploid formation on cell density where efficient mating was predominantly observed in cell concentrations higher than 5 million cells/ml, which coincided with cell distances permissive for mating (Figure 5A). At the same time we could observe a pronounced growth phase taking place in parallel with diploid formation (Figure 5B), giving support to the finding that there was indeed a variety of different phenotypes (mating and growing cells) occurring at the same time in the same culture. Figure 5C visualizes the cell densities used in the measurements for comparison and Figure S7 in Text S1 shows the simulated levels of **α**-factor under these conditions, with representation of **α**-factor distribution and Bar1 activity distribution for one selected cell density.

### Bar1 enables population growth coordinated with high mating efficiency and its absence leads to global cell cycle arrest upon pheromone stimulation

Our results suggested that Bar1 acts by restricting the activity of **α**-factor to sites, where successful mating is possible, leaving the remaining areas free for continued growth.

**Figure 6:**
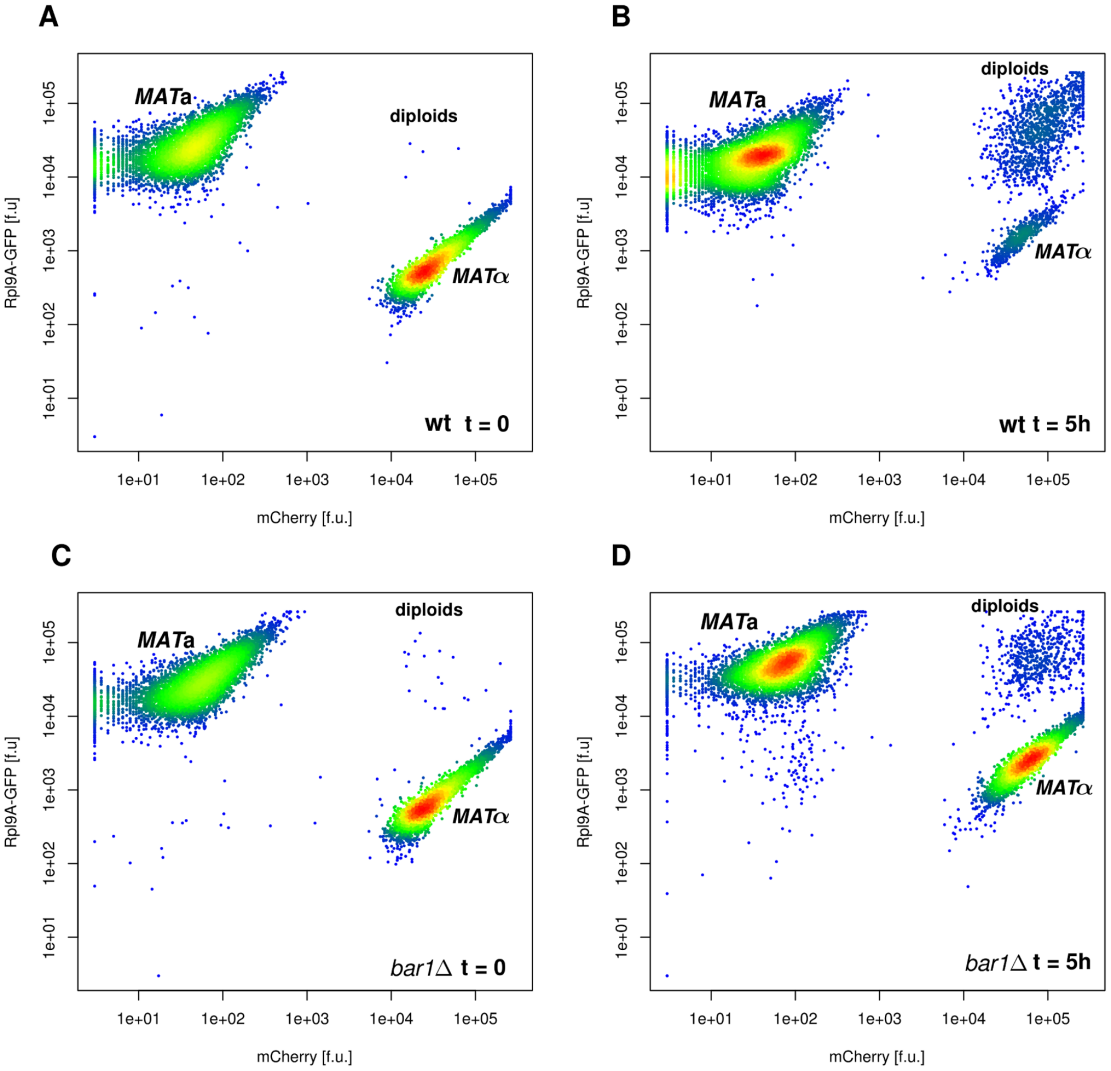
Flow cytometry has been used to quantify the fractions of diploids and *MAT*a or *MATα* haploids in the yeast cultures. *MAT* **a** and *MAT* ***α*** carried constitutively expressed GFP and mCherry constructs, respectively (Rpl9A-GFP and mCherry induced from the *TDH3* promoter). We observed a switch from many cells carrying either of the two constructs at the initial time point (A, C) to a large subpopulation carrying both constructs (B,D) after 5 hours. Density is indicated by colors from blue to red, two biological replicates of 10.000 cells each.

**Figure 7:**
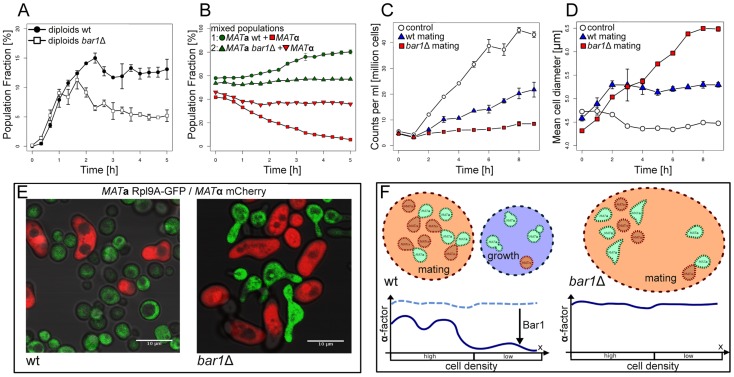
The two haploid strains and formed diploids were tracked using flow cytometry of cultures initially containing mixtures of *MAT*a Rpl9A-GFP and *MAT*α mCherry cells. (A) The population fraction of diploids in wild type and *bar1Δ* tracked over time (compare Figure S1 in Text S1, n = 20.000). (B) The population fraction of the two haploid cell types in wild type and *bar1Δ* cultures tracked over time (n = 20.000). (C) Average cell counts for the mixed populations in wild type and *bar1Δ* (n = 6). (D) Average cell diameters for the mixed populations in wild type and *bar1Δ* (n = 6). (E) Phenotypes of mixed cultures initially containing *MAT* **a** Rpl9A-GFP and *MAT* **α** mCherry after 26 hours. Images are overlays of the bright field, mCherry and GFP channels. (F) Illustration of the proposed mechanism for the local coordination of growth and signaling by Bar1.

In order to test the validity of this prediction and its dependency on Bar1 we observed mating between *MAT* **a** cells (here marked with Rpl9A-GFP) and *MAT* **α** cells (marked with mCherry) and quantified their growth rates with FACS analysis (Figure 6) and OD measurements in wild type and in *bar1Δ* populations during incubation (Figure 7).

Mating rates were quantified by the rate of diploid formation. To measure the diploid formation rate, we used flow cytometry for mixed populations of *MAT* **a** and *MAT* **α** (where we took care to not destroy extracellular gradients before the actual measurement) to quantify the fractions of *MAT* **a**, *MAT* **α** and *MAT* **a**/**α** diploids over time for a fixed number of cell counts (Figure 7A). We found no difference in the rate of diploid formation between wild type and *bar1Δ* cultures before completion of the first cell cycle (<120 min). This observation is in agreement with our results that positive effects on the perceived pheromone gradients require higher cell densities (Figure 4A,B). However, after passing the first cell cycle, the relative fraction of diploids is clearly larger in the wild type cultures than in the mutant, consistent with the general view that Bar1 activity helps to reveal the position of mating partners [3], [13], [14]

Looking at population growth during mating, we found strong differences between wild type and *bar1Δ* cultures (Figure 7B). For *bar1Δ* cultures, the global activation of the pheromone response in effectively all *MAT* **a** cells of the population led to an almost complete loss of population growth (Figure 7B,C). This also caused a characteristic population phenotype with many pheromone-stimulated *MAT* **a** cells being significantly larger than normal *MAT* **a** cells and showing multiple mating projections (Figure 7E,F). This phenotype was never encountered in unperturbed wild type mixtures of *MAT* **a** and *MAT* **α** cells, but could be induced by swirling them rapidly to inhibit cell fusion (Figure S8 in Text S1). Thus, this phenotype appears associated with induction of pheromone response *in vivo* under conditions where a cell cycle arrest has been induced but successful mating is inhibited.

Wild type cultures exhibited significant growth on the population level despite the higher rate of diploid formation and a normal phenotype of *MAT* **a** cells (Figure 7B,D–E).

The effect of Bar1 secretion on haploid growth rates was even more prominent when looking at the *MAT* **a/** *MAT* **α** ratio in the population (Figure 7C). There is no known secretion of an extracellular protease described for *MAT* **α** cells equivalent to Bar1. Co-cultured wild type *MAT* **a** cells strongly outperform *MAT* **α** cells in growth during mating to an extent that within 5 hours *MAT* **a** is the predominant haploid cell type in the population. This cannot be observed in *bar1Δ* background where the *MAT* **a/** *MAT* **α** ratio remains constant, presumably because both haploid cell types are equally inhibited in growth. In summary, secretion of Bar1 enables a high mating rate on a population level, but also strongly optimizes the population growth rate by avoiding unnecessary cell cycle arrest when mating is improbable (Figure 7F).

**Figure 8:**
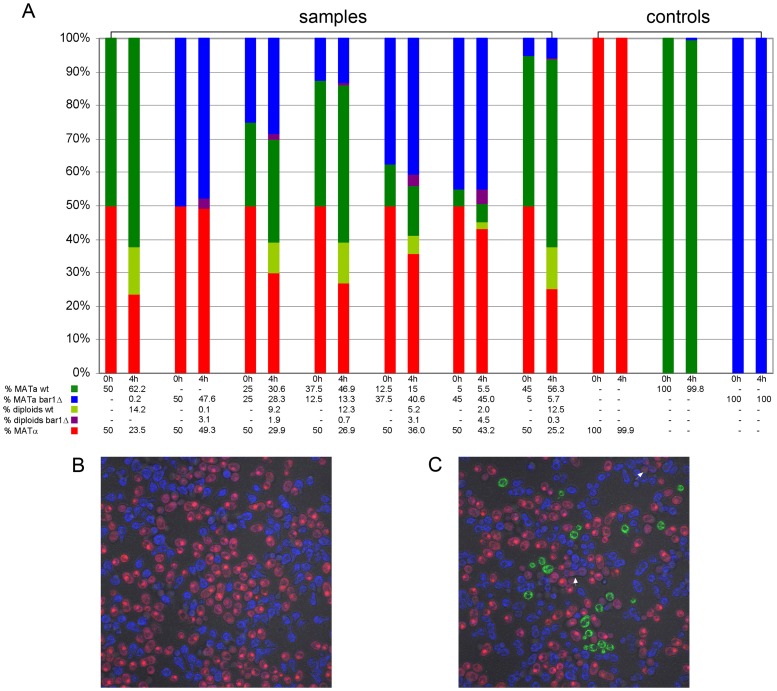
Do wild type and mutant cells influence each others' mating success? (A) Mating in mixed cultures initially containing *MAT* **α** cells (marked with mCherry) and varying fractions of wild type *MAT* **a** cells (marked with Rpl9A-GFP) and *MAT* **a** *bar1Δ* cells (marked with RPl9a-TagBFP2) after 4 h. (B) Confocal merged image of a mixed culture initially containing 50% *MAT* **α** and 50% *MAT* **a** *bar1Δ* cells. (C) Confocal merged image of a mixed culture containing 50% *MAT* **α** cells, 45% *MAT* **a** *bar1Δ* cells and 5% wild type *MAT* **a** cells. Some mating events are marked with white arrows. Each percentage represents the mean of two technical replicates with 10.000 counted cells each. Note that colors in the microscopic images and in the table (A) concur.

In order to further validate our findings about the role of Bar1 in the mating process, we mixed *MAT* **α** cells with different ratios of wild type and *bar1Δ MAT* **a** cells. We measured the amount of haploid and diploid cells after 4 h of incubation by FACS analysis (Figure 8). For labeling of *MAT*α cells we again used mCherry, whereas *MAT* **a** *BAR1* wild type cells were labeled with Rpl9a-GFP and *bar1Δ MAT* **a** cells with Rpl9a-TagBFP2. For equal ratios of wild type cells of both mating types at time 0 h we obtained a diploid fraction of 14% at time 4 h (leftmost columns). However, *MAT* **α** cells assumed about 23% while *MAT* **a** cells reached more than 62% of the total population, again supporting the observation that a part of the *MAT* **a** population engages in mating and other cells continue to grow.

For equal ratios of wild type *MAT* **α** and *bar1Δ MAT* **a** at start, we obtained only 3.1% diploids and roughly equal ratios of the haploid cells after 4 h. This is in agreement with the view that essentially all cells stop growing and start to prepare for mating. Microscopic imaging confirms that all cells are shmooing under this condition (Figure 8B). When mixing 50% of *MAT* **α** with different ratios of wild type *bar1Δ MAT* **a** cells, we obtain diploid cells of both types at ratios as could roughly be expected from the mixes with either wild type or mutant *MAT* **a**. Strikingly, for small ratios (5%) of wild type *MAT* **a** cells at 0 h, we see that *bar1Δ MAT* **a** mate more frequently (4.5% of total population) than in pure mutant mixes (3.1%), indicating that they profit from the Bar1 secreted by wild type *MAT* **a** cells. Figure 8C shows a microscopic image of a mixture of 5% wild type *MAT* **a** and 45% *bar1Δ MAT* **a** cells together with 50% *MAT* **α** at the beginning. Here, the *bar1Δ MAT* **a** cells exhibit clearly lower levels of shmooing compared to the 50%/50% mix in Figure 8B. A few successful mating events leading to diploids are indicated by white arrows.

Again, the experiments mixing wild type cells with cells not secreting Bar1 – the *bar1Δ* or cheater cells – confirm the role of Bar1 which is even supportive for those cells not actively secreting it, but experiencing its effect on **α**-factor levels and gradients.

## Discussion

The combination of spatial modeling and a series of in vivo experiments employing images of mating cells allowed us to quantitatively describe the distribution of the pheromone **α**-factor in the intercellular space and the role of the protease Bar1 in shaping the pheromone pattern. We found that secretion of Bar1 is a highly cooperative mechanism. Haploid *MAT* **a** cells secrete individually small quantities of Bar1 molecules. These few molecules are quickly distributed across the population by diffusion and generate Bar1 activity that strongly influences the diluted distribution of **α**-factor. For example, a low cell density of about 10^6^ cells/ml induces a degradation rate of **α**-factor in the range of 10^−3^ molecules per second which corresponds to almost no degradation of **α**-factor, however, high cell densities can create substantial degradation by the Bar1 activity where single **α**-factor molecules are cleaved within a second after secretion (compare Text S1). Thus, individual *MAT* **a** cells need to secrete only very small numbers of Bar1 protease. The global concentration of Bar1 appears to be fine-tuned to ensure a highly informative **α**-factor distribution. This effect is based on the interplay of the secretion of **α**-factor, its diffusion, the activation of Bar1 transcription and **α**-factor degradation by Bar1. Thus, we observed steep gradients in the distribution of **α**-factor only in high densities of *MAT* **a** cells where the joint degradation by Bar1 limits **α**-factor diffusion sufficiently. Our conclusions differ from what has been shown in previous publications where the effect of Bar1 is demonstrated either completely theoretical for one or a few cells [13], [14], [16] or experimentally for an artificial setup in a microfluidic device without mating partner [15]. First, we observed little to no effect of Bar1 in a setting with only few cells since the Bar1 activity is insufficient to degrade **α**-factor before it diffuses a large distance. Our results also indicate that Bar1 rather acts on the level of subpopulations than on a few individual cells. Second, we also did not observe an effect reported earlier that Bar1 secretion leads to “self-avoidance” [15]. Third, we modeled and experimentally quantified the induction of Bar1 due to stimulation with **α**-factor (compared to constant Bar1 levels as in [13] or constant secretion as in [14], [15]).

Remarkably, the simple circuit of pheromone secretion and degradation by jointly secreted Bar1 is able to produce highly dynamic behavior in yeast populations. In general, the **α**-factor concentration profile recovers the regions where mating has a high chance of success, but quickly drops at all other regions, thus allowing cells to continue growth when there is only little chance for successful mating (see Figure 7E, F). This is further regulated by a stimulated Bar1 production, which depends on the extracellular **α**-factor concentration. Stimulation of Bar1 secretion might be a strategy to adapt the zone of influence of **α**-factor to varying cell densities and numbers of *MAT* **α** cells in the population. Cells will react to high **α**-factor concentrations with strong secretion of Bar1, which culminates in a steady state permissive for efficient mating (also see Text S1 and Movie S1).

A lack of Bar1 in mixed haploid populations has crucial influence on the population phenotype since it leaves many cells in a prolonged cell cycle arrest in G1 phase along with activation of the pheromone response. Moreover, the temporally extended stimulation leads to larger cells with many mating projections. This result is further supported by the observation that *MAT* **a** cells strongly outperform *MAT* **α** cells in growth, an effect depending on Bar1. Thus, Bar1 is beneficial for growth as well as diploid formation because it enables continued growth for large parts of the population, but it also provides an enhanced ability to interpret the extracellular pheromone signal at sites where many cells cluster into locally dense subpopulations. While the gradient-enhancing effect of Bar1 has been reported before, we additionally connect it to the requirement of high local cell densities [13]. This indicates that the gradient enhancing ability of Bar1 has evolved to take place selectively in dense yeast populations and is not a treat of individual yeast cells in large volumes.

Our observations suggest that the yeast populations segregated into a spatial pattern with localized regions of diploid formation and other regions of continued growth. As a consequence, the entire population is divided into two different work programs. Both of those programs are performed in parallel and Bar1 is sufficient to induce this separation by forming a locally varying **α**-factor pattern. Depending on the overall cell density, the locations reserved for mating show high local concentrations of **α**-factor, whereas the locations reserved for growth show negligible concentrations (compare Figures 5 and 7). However, one may wonder how this cooperation arose during evolution since cheater *MAT* **a** cells not producing Bar1 can also easily exploit it. A possible explanation lies within the spatial structure of the population, since the fitness benefit conferred by Bar1 is locally restricted. Haploids can only arise from a small set of initial spores, which form subpopulations by budding and mating type switching. As a consequence, cells profiting from the collective secretion of Bar1 are likely to be genetically related. Under these circumstances there is indeed evidence that cooperation can be conserved during evolution [23], [24]. Our experiments show that cheater cells not producing Bar1 can indeed profit from the Bar1 secreted by non-cheater wild type *MAT* **a** cells in a mixed population (compare Figures 7 and 8). However, the non-cheater cells consistently outperform the cheater cells in mating as well as growth, indicating that the non-cheater cells maintain an advantage even in a mixed population of cheaters and non-cheaters.

The analyzed regulatory circuit of combined pheromone and protease secretion is not only observed in *Saccharomyces cerevisiae*, but is also found in other fungi [25]–[27]. Furthermore, a similar mechanism is known in *Dictyostelium discoideum* secreting phosphodiesterase (PDE) during detection of cAMP [28], [29]. Taking this into consideration, the described mechanism might be a general strategy to separate a cell population into subpopulations with different transcriptional programs.

Our methodology of quantifying the distribution of extracellular morphogens in the absence of direct measurement also has potential applications in other problems of cellular communication and pattern formation. A reduction from the computationally very expensive 3D problem (especially for parameter estimation) to an integrated 2D problem is feasible for any cells that sediment to the bottom of the containing volume under non-agitated conditions. However, this computational tool could be used to model the behavior of any culture in a non-moving liquid film such as on the surface of fruits or any controlled fermentation such as wine or beer production where the liquid is kept still for some time. This makes the method applicable for clinical research as well since biofilm formation involving quorum sensing is a major complication when fighting bacterial infections [30], [31]. Here, it might be helpful to quantify the distribution of quorum signals in order to find possible ways to optimally disrupt the system.

## Materials and Methods

### Used strains and constructs

Wild type *MAT* **a** reporter strains used in this study are Fus1-GFP and Rpl9A-GFP. They are based on BY4741 (*MAT* **a** *his3Δ1 leu2Δ0 met15Δ0 ura3Δ0*) and part of the yeast GFP collection [32]. The *MAT* **α** reporter strain expressing mCherry under control of the TDH3 promoter (*MAT* **α** *can1Δ STE2pr-SpHIS5 lyp1Δ::STE3pr-LEU2 his3Δ1 leu2Δ0 ura3Δ0 met15Δ hoΔ0::TDH3pr-mCherry-NATMX4*) was a friendly gift of Alexander DeLuna [33]. As mutant *MAT* **a** reporter strains we used two different strains: *bar1Δ* Fus1-GFP and *bar1Δ* Rpl9a-TagBFP2. The first mutant was created by deletion of the BAR1 gene in the Fus1-GFP strain mentioned above, the second was cloned by tagging of the Rpl9a Gene with Tag-BFP2 in the BY4741 *bar1Δ* strain. Rpl9a tagging was used because of the high expression level and also since it is not known to be involved in the mating process. Yeast strains were cultivated at 30°C in synthetic medium (0.17% yeast nitrogen base without amino acids, 0,5% ammonium sulfate, 2% glucose, 55 mg/l adenine, 55 mg/l L-thyrosine, 55 mg/l uracil, 20 mg/l L-arginine, 10 mg/l L-histidine, 60 mg/l L-isoleucine, 60 mg/l L-leucine, 40 mg/l L-lysine, 10 mg/l L-methionine, 60 mg/l L-phenylalanine, 50 mg/l L-threonine and 40 mg/l L-tryptophane). *BAR1* deletions in *MAT* **a** reporter strains were inserted by homologous integration of a *URA3* cassette in the *BAR1* locus (*bar1Δ0::URA3*). PCR amplification of the *URA3* cassette from plasmid template pESC-Ura (Stratagene) was done by sequential amplification with the primer pairs 1/2 and 3/4 shown in Table 1.

**Table 1:** PCR primer for BAR1 deletion.

Name	Sequence	Use
Primer 1	5′-GAAGGGTCATATAATGTCGCGCGTTTCGGTGATG-3′	PCR 1
Primer 2	5′-CTCCAGATTTCTTAGTTTTGCTGGCCGC-3′	PCR 1
Primer 3	5′-GGTTCGTATCGCCTAAAATCATACCAAAATAAAAAGAGT GTCTAGAAGGGTCATATAATG-3′	PCR 2
Primer 4	5′-GACTATATATTTGATATTTATATGCTATAAAGAAATTGTA CTCCAGATTTCTTA-3′	PCR 2

This was followed by transformation and selection on agar plates with synthetic medium lacking uracil. Verification of the *BAR1* deletion was done with a physiological assay based on growth inhibition by **α**-factor pheromone [34].

The *bar1Δ* Rpl9a-TagBFP2 reporter strain was cloned by PCR amplification of a TagBFP2 loxP-Ura3-loxP transformation cassette with primer pairs 5 and 6 from Table 2. As PCR template we used vector EKP232. EKP 232 was cloned by ligation of *TagBFP2* into PstI site of pUG72 [35].

**Table 2:** PCR primer for Rpl9a –TagBFP2 tagging.

Name	Sequence
Primer 5	5′-GGTATCTACGTTTCTCACAAGGGTTTTATTACTGAAGATTT AGGAGCAGGTGCTGG-3′
Primer 6	5′-CTGCTACTTTAAAGAAAATGTCACAAAATCAAATAAAAAGC GCATAGGCCACTAGTGGATCTG-3′

The qVenus expressing strain under control of the Bar1 promoter (Bar1pr-qVenus) was cloned by using the plasmid pSP 34 from Serge Pelet [20]. Bar1 promoter region [−500 bp] was amplified from genomic DNA (strain BY4741) by PCR using primer pair 9 and 10 as well as the first 51 bp of the *BAR1* gene using primer pair 7 and 8 shown in Table 3. PCR product of promoter and gene were mixed and used as template for a fusion PCR with a PmlI restriction site between promoter and gene. Included in forward and reverse primer were restriction sites for SacI and PstI respectively. The fusion PCR product was ligated into SacI/PstI site of pSP 34 resulting in plasmid EKP252. Plasmid EKP252 was linearized using PmlI restriction enzyme and used for transformation and homologous integration into the *BAR1* locus under preservation of the *BAR1* gene. Positive clones were selected in minimal medium lacking leucine, **α**-factor induced qVenus expression was controlled microscopically, and wild type Bar1 activity was verified by a physiological assay based on growth inhibition by **α**-factor pheromone [32] and comparison with Bar1 wild type and deletion strains.

**Table 3:** PCR primer for pBar1-qVenus reporter strain cloning.

Name	Sequence	Use
Primer 7	5′- GGAGCTCGCGCGAAACTCGCCAA -3′	*BAR1* gene
Primer 8	5′- CATCGACACGTGTCTAGAAGGGTC -3′	*BAR1* gene
Primer 9	5′- GACACGTGTCGATGAGTCCTTAAG -3′	promoter
Primer 10	5′- CCTGCAGTATATGACCCTTCTAG -3′	promoter

### Confocal microscopy and data analysis

Microscopic images were acquired with an inverted FluoView 1000 microscope (Olympus, Tokio, Japan) equipped with a 60× (1.2 N.A) water-immersion objective and a climate chamber (Tokai Hit, Japan). GFP was excited with a 488 nm argon laser and mCherry with a 559 nm laser diode. Fluorescence emission was detected in the range 500–545 nm and 570–670 nm, respectively. The Bar1pr-qVenus construct was excited with 515 nm and detected between 530 nm and 630 nm, for Rpl9a-TagBFP2 we used 405 nm excitation and as detection range 425–475 nm. For mating experiments, *MAT* **a** Fus1-GFP wild type and *bar1Δ* reporter strains as well as the *MAT* **α** reporter strain (TDH3pr-mCherry), were cultivated to mid logarithmic phase and mixed equally. Mating was followed over indicated time periods microscopically while microscopic samples were kept in cultivation medium at 30°C.

Image acquisition for **α**-factor calibration curves was done with synchronized cultures of Fus1-GFP wild type and *bar1Δ*. Cultures were synchronized in G1 phase by elutriation with a Beckman Coulter JE-5.0 elutriation system. Synchronized cells were incubated with **α**-factor pheromone for 3 hours at 30°C. Afterwards cells were spinned down on the surface of a glass bottom dish (MatTek Corporation, Ashland, US) by centrifugation at 100× g using self-built accessories. For Fus1-GFP wild type, **α**-factor pheromone concentrations in the range between 0 µM–100 µM were used and for *bar1Δ* we used 0.1 nM - 1 µM. Mean fluorescence intensity of Fus1-GFP was analyzed as described in the Computational Techniques (see Text S1). For validating the employment of Fus1-GFP as proxy for the mating response pathway we used a strain expressing qVenus under control of the Bar1 promoter in *BAR1* wild type background. Non-synchronized cells were incubated with **α**-factor pheromone as described for Fus1-GFP *BAR1* wild type cells and analyzed in the same way. A comparison of the results is shown in Figure S5 in Text S1.

### Growth curves and FACS analysis

Growth of equally mixed *MAT* **a** and *MAT* **α** reporter strains, as well as a haploid control strain was analyzed by measuring optical density at 600 nm with a Photometer (Eppendorf Bio Photometer plus) and in parallel by analysis of cell number and cell size distribution with a cell counter (Casy Counter TTC, Schärfe System). Yeast cells were incubated in a water bath at 30°C without shaking. In time steps of 15 min samples were removed from the water bath, vortexed, appropriately diluted, and analyzed in duplicate. To quantify the amount of haploid *MAT* **a** and *MAT* **α** cells, of diploid cells or of cells within the mating process, we measured fluorescence intensities for GFP and mCherry of 10.000 living cells of each sample by FACS analysis taking advantage of the fluorescence of *MAT* **a** Rpl9A-GFP and *MAT* **α** mCherry in a BD FACS AriaII cell sorter (Becton Dickinson, Franklin Lakes, NJ), equipped with a 488 nm and a 561 nm laser with filter sets for GFP (525/50 BP, 505LP) and for mCherry (610/20BP, 600LB). Cultures were incubated in a water bath at 30°C without shaking. In 20 min time steps, duplicate samples were removed from the water bath, mixed vigorously, diluted in PBS and FACS analyzed. Gates for *MAT* **a**, *MAT* **α** and diploids were set by hand identifying the cell types as shown in Figure 6.

### Analyzing the influence of the average cell distance on the mating process

As proof of the model prediction we performed a mating experiment with different cell densities. *MAT* **α** TDH3pr-mCherry and *MAT* **a** RPL9a-GFP *BAR1* wild type cells were grown in SD medium to mid log phase. Cells were diluted in SD medium and cell numbers were adjusted to 10·106 cells/ml by measuring the cell number with a CasyTTC cell counter. *MAT* **α** and *MAT* **a** cells were mixed 1∶1 and diluted in SD medium in following concentrations: 10·106, 5·106, 2.5·106, 1·106, 0.5·106 cells/ml. 2 ml aliquots of the diluted cultures were incubated at 30°C in Petri dishes with a diameter of 36 mm (Falcon), in order to get an average monolayer of cells after sedimentation. In time steps of 15 min one Petri dish of each cell dilution was removed from the incubator, cells were re-suspended in the medium by intensive pipetting and 400 µl of the cultures were mixed with 400 µl PBS supplemented with CaliBRITE APC Beads (BD Biosciences #340487). The samples were analyzed by FACS. APC Beads were recorded with 640 nm excitation and 670/41BP filter, *MAT* **a** Rpl9a-GFP and *MAT* **α** mCherry reporter strains as mentioned above. APC beads were gated and used as internal standard. In each sample the number of cells corresponding to a fixed number of 90000 APC beads was analyzed, giving not only the relative amount of haploids and mating events but also the growth behavior of the components of the mixed culture (results of the experiment are shown in Figure 5).

### Influence of varying amounts of *MAT*a *bar1Δ* cheater cells in mixed cultures with *BAR1* wild type cells

To analyze the influence of *bar1Δ* cheater cells in mating mixtures we used *MAT* **a** Rpl9a-TagBFP2 *bar1Δ* reporter strain, together with the already introduced *MAT* **a** and *MAT* **α** reporter strains. The three strains were grown in SD media to mid logarithmic growth phase and diluted in SD media to 1·10^7^ cells/ml. Several mating-with-cheaters-mixtures were prepared as shown in Table 4.

**Table 4:** Ratios for mating mixtures with cheater.

Cell Type	0% Cheaters	100% Cheaters	50% Cheaters	25% Cheaters	75% Cheaters	90% Cheaters	10% Cheaters
*MAT* **α** TDH3pr-mCherry	2 ml	2 ml	2 ml	2 ml	2 ml	2 ml	2 ml
*MAT* **a** RPL9a-GFP Bar1 wt	2 ml		1 ml	1.5 ml	0.5 ml	0.2 ml	1.8 ml
*MAT* **a** RPL9a-TagBFP2 *bar1Δ*		2 ml	1 ml	0.5 ml	1.5 ml	1.8 ml	0.2 ml

### Computational methods

The images were analyzed with CellID [36] to extract mating type, fluorescence activity, as well as position, size and shape of the cells. These data were transferred to a computational domain (see Figures S2 and S3 in Text S1 for details). From this computational domain a triangular mesh was generated using Gmsh [37] that can be used by various RD toolboxes. Here, we used the open source Toolbox DUNE [38] to solve the stationary as well as time-dependent equations by a finite element method with high accuracy [39], [40]. In Supporting Text S2 we systematically compare 2D and 3D simulations to account for the fact that images are taken in 2D, while diffusion and mating happen in 3D (see Figures S9, S10, S11 in Text S2). We found that the maximum difference between 2D and 3D in the simulated **α**-factor distribution was below 5%. Therefore, for the parameter fit the 2D solution was used, which resulted in a major speed-up.

## Supporting Information

Movie S1 **Dynamic simulation of an experimental mixed haploid population.** In the movie generated from confocal images to the left, images are overlays of the bright field channel, GFP channel (*MAT* **a** Fus1-GFP) and mCherry channel (*MAT* **α** mCherry). The simulated **α**-factor distribution is shown on the right. In the simulation red indicates *MAT* **α** cells and white *MAT* **a** cells. Each second in the movie corresponds to 5 minutes in real time. Due to the delay in the pheromone dependent induction of Bar1, **α**-factor will initially accumulate to high concentrations, which is counteracted by a strong degradation of **α**-factor caused by Bar1 induction after 30 minutes. Finally, the **α**-factor level rises again due to the loss of Bar1 induction to its steady state value. It should be noted that shmoo formation seems to take place shortly after the final stable gradient has been formed, indicating a good timing between regulation of the extracellular **α**-factor distribution and mating. The movie requires the Xvid codec (http://www.xvid.org).
(AVI)

Text S1 **Computational techniques.** Detailed description of the image based quantification of pattern formation. Computational methods and equations are described in detail as well as the employed tools. Additional microscopic images are shown in Figures S1 and S8. The computational domain and generated meshes are explained in Figure S2 and S3. The calibration curves referring to Figure 1 can be found in Figures S4 and S5. For experimental data and simulations showing the interdependence of mating events and cell density see Figures S6, S7 and Table S2. Model assumptions and parameters from recent works are listed in Table S1.
(PDF)

Text S2 **Comparison between 2D and 3D model formulations.** Computational quantification of the relation between the 2D and 3D model for several arrangements of *MAT* **α** and *MAT* **a** cells are shown in Figures S9 to S11. The transformation of important quantities is listed in Table S3 and computational results are shown in Table S4.
(PDF)
