# Supplementary material for: Yeast Mating and Image-Based Quantification of Spatial Pattern Formation
Source: PLoS Comput Biol. 2014 Jun 26;10(6):e1003690. doi: 10.1371/journal.pcbi.1003690 (PMC4072512; doi:10.1371/journal.pcbi.1003690)
Supplement: Text S1 — Computational techniques. Detailed description of the image based quantification of pattern formation. Computational methods and equations are described in detail as well as the employed tools. Additional microscopic images are shown in Figures S1 and S8. The computational domain and generated meshes are explained in Figure S2 and S3. The calibration curves referring to Figure 1 can be found in Figures S4 and S5. For experimental data and simulations showing the interdependence of mating events and cell density see Figures S6, S7 and Table S2. Model assumptions and parameters from recent works are listed in Table S1. (PDF) [file pcbi.1003690.s002.pdf]

Supporting Text S1 for Yeast mating and image-based quantification of spatial pattern formation

Christian Diener[1,2*], Gabriele Schreiber[1*], Wolfgang Giese[1*], Gabriel del Rio[2], Andreas Schröder[3], Edda Klipp [1*]

[1*] Theoretische Biophysik, Humboldt-Universität zu Berlin,
Invalidenstr. 42, 10115 Berlin, Germany

[2] Instituto de Fisiología Celular, Universidad Nacional Autónoma de México
S/N Ciudad Universitaria, 04510 México D.F, México

[3] Department of Mathematics, University of Salzburg,
Hellbrunnerstr. 34, 5020 Salzburg, Austria

*Correspondence to: edda.klipp@biologie.hu-berlin.de

Contents

Computational Techniques 2
- Image analysis with CellID to identify cell type and location . . . 2
- Construction of the computational domain for simulations . . . 2
- Dynamic model equations . . . 3
  - Boundary and initial conditions . . . 3
- Model reduction and stationary distributions . . . 4
- Calibration and Optimization . . . 5
  - Calculating the local α-factor concentrations . . . 5
  - Estimating the Bar1 activity kinetics . . . 6
  - Estimating the average α-factor secretion rate . . . 7
- Analysis of α-factor distributions . . . 8
- Numerical methods . . . 9
  - Implementation . . . 9

Figures S1 to S8 11

Tables S1 and S2 17

Computational Techniques

We established an efficient workflow between experiment and mathematical model and automatized major parts of the whole process. In the following, we describe the procedure from analyzing microscopic images to finally visualize the calculated spatial patterns of the mating pheromone distribution. In particular, we introduce the methods for the automatic identification of mating types on images as well as the transformation of microscopic pictures to the corresponding computational domains, which are represented as finite element meshes. Furthermore, a detailed explanation of the mathematical model equations as well as the boundary conditions is given. The presented computational approach allows us to estimate $\boldsymbol{\alpha}$-factor distributions by calculating pheromone distributions and fluxes for various spatial cell arrangements in the experimental setup.

Image analysis with CellID to identify cell type and location

Images were generated using the Olympus Software as described in the main text (see Materials and Methods) and analyzed with CellID [1]. In order to prepare them for analysis, all z positions starting from the focal plane to the most extreme out-of-focus coordinate (thus, the upper half of the z-stack) were projected over the z-axis via an average intensity projection. This was done in order to obtain the required out-of-focus images with low noise levels. Since far out-of-focus images in the fluorescent channels generally showed a strong autofluorescence only the three slices closest to the focus were used for an average intensity z-projection here. No other modifications were applied to the images prior to analysis. Figure 1 and Figure 3 (see main text) were generated by an overlay of the bright field, GFP and mCherry channel. Here the contrast was adapted linearly to aid visibility (originals shown in Figure S1). However, no contrast or pixel value modification was ever applied to images used for analysis or quantification.

The generated projections were consecutively analyzed using VCellID and a modified version of CellID [2]. Here, the modification of CellID led to additional text file output containing the individual boundaries for each detected cell along with a list of all interior pixels and by-pixel fluorescence values contained within an area extending the cell boundary by 3 pixels. Parameters for CellID were chosen within VCellID to maximize the fraction of detected cells. The output was further analyzed with the statistical scripting environment R [3].

Using CellID output, we expressed the x and y locations in micrometers. For each cell i the fluorescence level (mCherry for $MAT\boldsymbol{\alpha}$ and GFP for $MAT\mathbf{a}$) was translated to the average fluorescence per pixel F_i with

$$F_i = \frac{F^i_{tot}}{n^i_{pix}} - F_{bg}. \tag{1}$$

Here, F^i_{tot} denotes the total fluorescence for a given cell with index i, n^i_{pix} the number of contained pixels and F_{bg} is the background fluorescence, which is calculated from the intensities of the pixels not contained in cells. Since $MAT\boldsymbol{\alpha}$ cells were labeled with a constitutive mCherry marker, cells with fluorescence value above four times the background in the mCherry channel were identified as $MAT\boldsymbol{\alpha}$ cells. All other cells were considered $MAT\mathbf{a}$ haploids.

Construction of the computational domain for simulations

We use real cell positions and activation levels to enable realistic simulations of the distributions of $\boldsymbol{\alpha}$-factor and Bar1 activity. To this end, individual cell boundaries were read from the output of the modified CellID and were transferred to polar coordinates based on the center of the cells. This information was used to approximate cell boundaries with a smoothing spline. Overlaps occurring after smoothing were resolved automatically. The generated cell shapes were used to build up the geometry and mesh for the finite element representation, for which meshing was performed within the Gmsh framework [4] and, thus, conforming to its geometry formats. The construction of the finite element mesh was started from a disk with a given radius chosen large enough such that it left sufficient space within the outer regions of the mesh. Cells were cut out of the disk according to the spline generated from the boundary pixels in order to yield a geometry reflecting the cell boundaries on the image. A unique boundary ID was assigned to each cell which was later used to identify the cell type ($MAT\mathbf{a}$ or $MAT\boldsymbol{\alpha}$). The resulting geometry was then used to generate a triangular mesh with the Frontal algorithm implemented in Gmsh (compare Figure S2). Refinement of the grid increased the accuracy of the approximate finite element solution. Since we were interested in obtaining high-accuracy solutions particularly in regions close to cells, the size of the generated triangles was adapted automatically in the vicinity of cells or at sites of complex geometry. This was achieved through the calculation of a local density map from the binned densities of all original boundary points returned by CellID with 128 bins in the x and y direction. A diameter size S

was then assigned to each point used in the geometry of the grid via the relation

$$S = \max\{\lambda_S \cdot e^{-\gamma D(x,y)}, \lambda_M\}, \tag{2}$$

where $D(x, y)$ is the local density of the point and γ, λ_S and λ_M were scaling parameters controlling the minimum and maximum target size. The generated diameter sizes for the triangles were imposed upon the Frontal algorithm to create a mesh that is able to represent the cell shapes. The resulting mesh was then exported and used for the following finite element simulations and parameter optimization.

Dynamic model equations

In the experimental set up described above the cells were put on a dish within a thin fluid layer of height h. To simulate the diffusion of $\boldsymbol{\alpha}$-factor and Bar1 during a given time period $[0, T]$ we establish a system of reaction-diffusion equations on a computational domain $\Omega \subset \mathbb{R}^2$ (see Figure S3). This domain represents the extracellular space in the view from above. The following system of partial differential equations describes concentrations, i.e. molecules per area. Note that the projection onto a two dimensional domain does not affect the diffusion constants. We consider free diffusion of $\boldsymbol{\alpha}$-factor and Bar1 in the extracellular medium and the degradation of $\boldsymbol{\alpha}$-factor by Bar1. The system of reaction-diffusion equations reads as:

$$\frac{\partial \alpha(\boldsymbol{x}, t)}{\partial t} = D_\alpha \Delta \alpha(\boldsymbol{x}, t) - k \cdot Bar1(\boldsymbol{x}, t) \cdot \alpha(\boldsymbol{x}, t) \quad \text{in } \Omega \times (0, T), \tag{3}$$

$$\frac{\partial Bar1(\boldsymbol{x}, t)}{\partial t} = D_B \Delta Bar1(\boldsymbol{x}, t) \quad \text{in } \Omega \times (0, T). \tag{4}$$

D_α and D_B denote the diffusion contants of $\boldsymbol{\alpha}$-factor and Bar1, respectively. The Laplace operator Δ denotes the second order derivative with respect to the spatial dimension. The reaction term on the right hand side of equation (3) effectuates a negative feedback from Bar1 on the $\boldsymbol{\alpha}$-factor concentration. Secretion and absorption processes are described via flux conditions (also called Neumann boundary conditions) as explained below. The $\boldsymbol{\alpha}$-factor absorption of each $MAT\mathbf{a}$ cell is measured by a boundary integral over the surface of the $MAT\mathbf{a}$ cells, which determines the secretion of Bar1.

Boundary and initial conditions

Since the interior of the cells is excluded from the computational domain, the boundaries also define cell walls (compare Figure S3). These boundaries are denoted by Γ_{α_i} and Γ_{a_j} for the i-th $MAT\boldsymbol{\alpha}$ cell and for the j-th $MAT\mathbf{a}$ cell, respectively, where an appropriate numbering is used. The outer boundary is denoted by Γ_{sur}.
The secretion of $\boldsymbol{\alpha}$-factor by the $MAT\boldsymbol{\alpha}$ cells is specified via Neumann boundary conditions, i.e. via the specification of the flux of the $\boldsymbol{\alpha}$-factor over the boundary, where a constant secretion of $\boldsymbol{\alpha}$-factor by each of these cells is assumed:

$$- D_\alpha \cdot \nabla \alpha(\boldsymbol{x}, t) \cdot \boldsymbol{n}(\boldsymbol{x}) = J_{\alpha_i} \quad \text{on } \Gamma_{\alpha_i} \times (0, T),\ i = 1, \ldots, N_\alpha. \tag{5}$$

N_α denotes the number of $MAT\boldsymbol{\alpha}$ cells. The vector $\boldsymbol{n}(\boldsymbol{x})$ is the unit outer normal with respect to the boundary and points into the cell interior. The other vector $\nabla\alpha(\boldsymbol{x}, t)$ is a spatial gradient of $\alpha(\boldsymbol{x}, t)$. Hence, the mathematical expression $-D_\alpha \nabla\alpha \cdot \boldsymbol{n}(\boldsymbol{x})$ is the flux of $\boldsymbol{\alpha}$-factor from the intracellular into the extracellular area, which is the secretion rate J_{α_i}. On the boundary of the $MAT\mathbf{a}$ cells the $\boldsymbol{\alpha}$-factor is detected. It is assumed that binding effects on the concentration of $\boldsymbol{\alpha}$-factor in the extracellular domain can be neglected. Hence, homogeneous Neumann boundary conditions are imposed:

$$- D_\alpha \cdot \nabla \alpha(\boldsymbol{x}, t) \cdot \boldsymbol{n}(\boldsymbol{x}) = 0 \quad \text{on } \Gamma_{a_i} \times (0, T),\ i = 1, \ldots, N_a, \tag{6}$$

with N_a the number of $MAT\mathbf{a}$ cells. Similarly, homogeneous Neumann boundary conditions are assumed for the surrounding, i.e. the exchange of $\boldsymbol{\alpha}$-factor between the computational domain and the remaining cell culture is in equilibrium:

$$- D_\alpha \cdot \nabla \alpha(\boldsymbol{x}, t) \cdot \boldsymbol{n}(\boldsymbol{x}) = 0 \text{ on } \Gamma_{sur} \times (0, T). \tag{7}$$

The $MAT\mathbf{a}$ cells are assumed to secrete the aspartyl protease Bar1 depending on the $\boldsymbol{\alpha}$-factor that is present on the cell surface. Bar1 expression depends on the average concentration of $\boldsymbol{\alpha}$-factor on the boundary of the considered cell I_{α_i} in the following manner:

$$I_{\alpha_i}(t) = \frac{1}{|\Gamma_{a_i}|} \int\limits_{\Gamma_{a_i}} \alpha(\boldsymbol{x}, t) dS, \ i = 1, \dots, N_a. \tag{8}$$

Secretion of Bar1 is induced by the resulting gene expression and, therefore, the Bar1 concentration in the vicinity of the cell wall should follow the same kinetics as the Fus1 expression which is obtained from the imaging experiments. Hence, the Bar1 concentration is given by the following Dirichlet boundary condition:

$$\begin{aligned} Bar1(\boldsymbol{x}, t) = k_0 + k_1 \frac{I_{\alpha_i}(t-\tau)^H}{I_{\alpha_i}(t-\tau)^H + EC_{50}^H} \\ \text{on } \Gamma_{a_i} \times (0, T), \ i = 1, \dots, N_a. \end{aligned} \tag{9}$$

The constant k_0 is the basal expression of Bar1 and k_1 is the maximal expression upon detection of $\boldsymbol{\alpha}$-factor, whereas τ is the delay that represents the time required for the processes from the pheromone pathway activation to the expression of Bar1. The coefficient H and the kinetic constant EC_{50} are the parameters of the Hill curve used for the fluorescence expression.

Note that Bar1 is neither absorbed nor secreted by the $MAT\boldsymbol{\alpha}$ cells. Thus, homogeneous Neumann boundary conditions are assumed:

$$\begin{aligned} & - D_B \cdot \nabla Bar1(\boldsymbol{x}, t) \cdot \boldsymbol{n}(\boldsymbol{x}) = 0 \\ & \text{on } \Gamma_{a_i} \times (0, T), \ i = 1, \dots, N_a. \end{aligned} \tag{10}$$

On the exterior boundary Bar1 we also assume Neumann boundary conditions for Bar1. To describe an equilibrium exchange with the environment due to the fast Bar1 diffusion:

$$-D_B \cdot \nabla Bar1(\boldsymbol{x}, t) \cdot \boldsymbol{n}(\boldsymbol{x}) = 0 \text{ on } \Gamma_{sur} \times (0, T). \tag{11}$$

Moreover, for the initial conditions we assume:

$$\begin{aligned} \alpha(\boldsymbol{x}, 0) &= \alpha_0(\boldsymbol{x}) && \text{in } \Omega, \\ Bar1(\boldsymbol{x}, 0) &= Bar1_0(\boldsymbol{x}) && \text{in } \Omega. \end{aligned} \tag{12}$$

Model reduction and stationary distributions

Since we measured the degradation of $\boldsymbol{\alpha}$-factor by Bar1 in the experiments, we found that the concentration of $Bar1(\boldsymbol{x}, t)$ and the degradation constant k cannot be estimated at the same time as they are strongly correlated in the parameter estimation results. Therefore, the reaction-diffusion equations (3),(4) for Bar1 were simplified by introducing the Bar1 activity $B(\boldsymbol{x}, t) = k \cdot Bar1(\boldsymbol{x}, t)$. This yields the equation system:

$$\begin{aligned} \frac{\partial \alpha(\boldsymbol{x}, t)}{\partial t} &= D_\alpha \Delta \alpha(\boldsymbol{x}, t) - B(\boldsymbol{x}, t) \cdot \alpha(\boldsymbol{x}, t) \\ & \text{in } \Omega \times (0, T), \end{aligned} \tag{13}$$

$$\frac{\partial B(\boldsymbol{x}, t)}{\partial t} = D_B \Delta B(\boldsymbol{x}, t) \text{ in } \Omega \times (0, T). \tag{14}$$

Note, that also the inhomogeneous boundary conditions have to be adapted:

$$\begin{aligned} & B(\boldsymbol{x}, t) = \tilde{k}_0 + \tilde{k}_1 \frac{I_{\alpha_i}(t-\tau)^H}{I_{\alpha_i}(t-\tau)^H + EC_{50}^H} \text{ on } \Gamma_{a_i}, \\ & i = 1, \dots, N_a, \end{aligned} \tag{15}$$

where $\tilde{k}_0 = k \cdot k_0$ and $\tilde{k}_1 = k \cdot k_1$. In the remainder, we use B instead of $k \cdot Bar1$ and assume that the $\boldsymbol{\alpha}$-factor secretion rates are proportional to the size of the $MAT\boldsymbol{\alpha}$ cells and hence, $J_{\alpha_i} = J_\alpha$ for all $i = 1, \dots, N_\alpha$. The term B also includes non-specific spontaneous degradation of $\boldsymbol{\alpha}$-factor. A Bar1 deletion ($bar1\Delta$) is simulated by reducing B to a constant with a constant Dirichlet boundary on all $MAT\mathbf{a}$ cells and an outer Neumann

condition. This results in a constant distribution of B. Thus, for a deletion we simply set $\tilde{k}_1 = 0$ and $\tilde{k}_0 = 10^{-3}$ as $\boldsymbol{\alpha}$-factor should show no significant spontaneous degradation on a scale of 15 minutes.

The steady state distribution of $\boldsymbol{\alpha}$-factor and Bar1 activity B is derived by omitting the time derivative on the left hand sides of equations (13) and (14). This leads to the following reaction-diffusion system

$$D_\alpha \Delta\alpha(\boldsymbol{x}) = B(\boldsymbol{x}) \cdot \alpha(\boldsymbol{x}) \qquad \text{in } \Omega, \tag{16}$$

$$D_B \Delta B(\boldsymbol{x}) = 0 \qquad \text{in } \Omega. \tag{17}$$

Also the boundary conditions are adapted to the stationary model. Hence, the time dependent boundary condition equation (9) becomes:

$$B(\boldsymbol{x}) = \tilde{k}_0 + \tilde{k}_1 \frac{I_{\alpha_i}^H}{I_{\alpha_i}^H + EC_{50}^H} \text{ on } \Gamma_{a_i},\ i = 1, \dots, N_a,$$

$$I_{\alpha_i} = \frac{1}{|\Gamma_{a_i}|} \int_{\Gamma_{a_i}} \alpha(\boldsymbol{x}) dS. \tag{18}$$

The remaining boundary conditions equations (5)-(7) and equations (10), (11) are exactly the same as in the instationary model.

Only the parameters $J_{\alpha_i}, \tilde{k}_0$ and $\tilde{k}_1$ have to be determined by parameter estimations from the microcopic data. The diffusion constants can instead be calculated from the protein properties since the medium we consider is only in the cell free space and thus is essentially an aqueous solution. The rates have been estimated by applying the Einstein-Stokes-Relation

$$D = \frac{k_B T}{6\pi \cdot \eta \cdot r}, \tag{19}$$

where k_B denotes the Boltzmann constant, T the temperature of the medium, η the viscosity of the medium and r the protein radius. The radius r is approximated from the density derived via the protein mass and applying the correction of Fischer et al. [5] to approximate the density of the protein from its mass:

$$\rho(M) = 1.41 + 0.145 \cdot \exp\left(\frac{M[kDa]}{13}\right) [g \cdot cm^{-3}]. \tag{20}$$

Converting that to $[kg \cdot cm^{-3}]$ and assuming a globular shape for the protein within the aqueous medium M with viscosity η_M the diffusion rate of a protein P is, thus, given by

$$D(P) = \frac{k_B T}{6\pi \cdot \eta_M} \cdot \left(\frac{750 \cdot M_P}{\pi \cdot \rho(M_P)}\right)^{-1/3}. \tag{21}$$

Substituting the masses by the ones known for $\boldsymbol{\alpha}$-factor and Bar1 results in effective diffusion rates of $361.48\mu m^2 s^{-1}$ and $104.63\mu m^2 s^{-1}$, respectively. The relevant temperature of the climate chamber was 30 °C.

Calibration and Optimization

The parameters $J_{\alpha_i}, \tilde{k}_0, \tilde{k}_1, EC_{50}^H$ and H of the mathematical model (equations (13)-(14)) were deduced from our experimental data on cell location and activation in three major steps. First, as a calibration, $bar1\Delta$ mutants were used to establish an unambigious relation between the expression of Fus1 and the $\boldsymbol{\alpha}$-factor concentration perceived by a cell. This relationship was fitted to a sigmoidal curve, and the relevant parameters $EC50$ and H were identified. In the second setup, we observed solely $MAT\mathbf{a}$ cells of wild type to estimate the activity of Bar1 due to supplied $\boldsymbol{\alpha}$-factor. We measured Fus1-GFP as indicator for Bar1 expression. The estimation employed solving a nonlinear ordinary delay differential equation with respect to an optimization problem. Finally, the model equations were solved in a spatial arrangement to determine the $\boldsymbol{\alpha}$-factor secretion rate. This was performed to obtain best model behavior and prediction of the spatial pheromone patterns for various cell arrangements. Details of the three steps are explained in the following.

Calculating the local α-factor concentrations

In order to quantitatively couple the solution of the reaction-diffusion system to the images we needed to relate the solution to the observable expression of Fus1. Since the model only predicts the local integrated $\boldsymbol{\alpha}$-factor concentrations and Bar1 activity we required a calibration relating the Fus1 expression level to the local $\boldsymbol{\alpha}$-factor

concentrations at the membrane of the cells. This was achieved by measuring the Fus1 expression levels via the fluorescence intensity of Fus1-GFP in $MAT\mathbf{a}$ $bar1\Delta$ cells over a range of different $\boldsymbol{\alpha}$-factor concentrations. Since Bar1 could not influence the concentration of $\boldsymbol{\alpha}$-factor in this genetic background, all cells were exposed to the same level of $\boldsymbol{\alpha}$-factor. Fus1 expression was quantified after 3 hours using confocal microscopy images. The individual GFP fluorescence of each cell was quantified using CellID and R as described above. Since it has been reported that the response behavior of the pathway follows a Hill curve, we fitted the fluorescence values to that functional form [6, 7]. This yielded a functional relationship between Fus1-GFP expression and local $\boldsymbol{\alpha}$-factor concentrations of the form

$$F_{obs} = F_{max} \cdot \frac{\alpha(t-\tau)^H}{\alpha(t-\tau)^H + EC_{50}^H} + F_{base}. \tag{22}$$

For a given $\boldsymbol{\alpha}$-factor concentration the distribution of fluorescence values showed a normal distribution (105-588 cells for each concentration). The fitting of the Hill curve was performed by optimizing the log-likelihood of the calculated curve under the data by iteratively using a Simulated Annealing run followed by a Broyden–Fletcher–Goldfarb–Shanno (BFGS) optimization (see Figure S5 for results). Significance was assessed using the F-test. For any given local $\boldsymbol{\alpha}$-factor concentration equation (22) provides a unique relationship between the concentration and the steady state Fus1-GFP fluorescence. The surface of a spherical $MAT\mathbf{a}$ cell is by a factor $2r_i$ bigger than the circumference of the cell. This has to be taken into account since our model employs integrated quantities over the z-axis. In order to ensure the same $\boldsymbol{\alpha}$-factor levels in the integrated model the observed average $\boldsymbol{\alpha}$-factor concentration has to be scaled by this factor. As a consequence, for a given observed Fus1-GFP expression we can derive the local $\boldsymbol{\alpha}$-factor concentration α_i in direct vicinity of the $MAT\mathbf{a}$ cell i from the observed Fus1-GFP fluorescence F_i in steady state as

$$\alpha_i = 2r_i \cdot EC_{50} \cdot \left(\frac{F_i}{F_{max} + F_{base} - F_i} \right)^{\frac{1}{H}}. \tag{23}$$

Because F_{base} is the autofluorescence of cells not expressing Fus1-GFP this quantity can also be quantified by the signal in the GFP channel of the $MAT\boldsymbol{\alpha}$ mCherry cells which do not carry Fus1-GFP.

Estimating the Bar1 activity kinetics

In the experimental setup for the estimation of the Bar1 activity only $MAT\mathbf{a}$ cells of the wild type are present and $\boldsymbol{\alpha}$-factor in given amounts is added to the cellular arrangement. Therefore, we assume that spatial inhomogeneities can be neglected. To estimate the parameters $\tilde{k}_0$ and $\tilde{k}_1$ in equation (15) we reduce the PDE system equations (13), (14) to an ordinary differential equation. Integration of equation (13) over Ω and application of Green's formula gives the continuity equation

$$\begin{aligned} \frac{d}{dt} \int_{\Omega} \alpha(\boldsymbol{x},t) dV = &- D_\alpha \int_{\partial\Omega} \nabla \alpha(\boldsymbol{x},t) \cdot \boldsymbol{n}(\boldsymbol{x}) dS \\ &- \int_{\Omega} B(\boldsymbol{x},t)\alpha(\boldsymbol{x},t) dV. \end{aligned} \tag{24}$$

Insertion of the boundary conditions (equations (5)-(7)) gives:

$$\frac{d}{dt} \int_{\Omega} \alpha(\boldsymbol{x},t) dV = \sum_{i=0}^{N_\alpha} \int_{\Gamma_{\alpha_i}} J_{\alpha_i} dS - \int_{\Omega} B(\boldsymbol{x},t)\alpha(\boldsymbol{x},t) dV. \tag{25}$$

Since there are no $MAT\boldsymbol{\alpha}$ cells in the experimental setup, equation (25) simplifies to:

$$\frac{d}{dt} \int_{\Omega} \alpha(\boldsymbol{x},t) dV = - \int_{\Omega} B(\boldsymbol{x},t)\alpha(\boldsymbol{x},t) dV. \tag{26}$$

The average $\boldsymbol{\alpha}$-factor concentration is $\bar{\alpha} = \frac{1}{|\Omega|} \int_{\Omega} \alpha(\boldsymbol{x},t) dV$ and it holds:

$$|\Omega| \frac{d}{dt} \bar{\alpha}(t) = -|\Omega| \bar{\alpha}(t) \bar{B}(t). \tag{27}$$

Furthermore, we deduce the average Bar1 activity from the Dirichlet boundary condition equation (15). This yields a nonlinear equation with delay of the form:

$$\frac{d}{dt}\bar{\alpha}(t) = -\bar{\alpha}(t)\left(\tilde{k}_0 + \tilde{k}_1 \frac{I_{\bar{\alpha}}(t-\tau)^H}{I_{\bar{\alpha}}(t-\tau)^H + (EC_{50})^H}\right). \tag{28}$$

This delay ODE can be solved for each of the different $\boldsymbol{\alpha}$-factor concentrations yielding $\boldsymbol{\alpha(t)} = \{\alpha_i(t)\}$, $i = 1, \ldots, N_c$. This leads to a predicted fluorescence $\mathbf{F}(t) = \{F_i(t)\}$ when plugging $\alpha(t)$ into equation (22). We fitted the parameters $\tilde{k}_0$, $\tilde{k}_1$ and τ to our experimental data by a maximum likelihood optimization using the probability distributions of the observed fluorescence of each of the N_c different experiments (see Figure S5). The fluorescence values were given by normal distributions $f_i(x, \mu_i, \sigma_i)$ measured at timepoint t_{obs} (147-746 cells for each concentration, 2458 cells in total). The resulting optimization problems is given by:

$$\begin{aligned} &\text{Find } \tilde{k}_0, \tilde{k}_1, \tau \text{ such that for} \\ &\mathbf{F}(t) = \{F_i(t_{obs})\} : \sum_{i=1}^{N_c} \ln f_i(F_i(t_{obs}), \mu_i, \sigma_i) \to \max. \end{aligned} \tag{29}$$

The ODE was solved by the fully adaptive implicit LSODA method and used during the optimization by again iterating Simulated Annealing and BFGS runs. Significance was assessed using an F-test. The fitted parameters were the steady state (stimulated) Bar1 activities and the delay time τ under the given number of cells in the population and the given volume.
The delay time τ was optimized here since this is the only time course optimization we perform ($\boldsymbol{\alpha}$-factor secretion parameter is fitted later to the steady state). The activation of the Ste12 transcription factor takes at least 5 minutes [6]. Considering that Bar1 also has to be transcribed, translated, modified and exported we do expect that cells will need more time to adequately react with an increased Bar1 secretion. The fitted value of $\tau \approx 30\,min$ is in good agreement with this consideration and our own observations that shmooing started later than half an hour after mixing of cultures.
However, since all cells secrete Bar1 the steady state Bar1 activity is dependent on the number, and possibly, size of the cells in the population. Since the overall Bar1 activity will scale linearly with the total amount of Bar1 secreted, we normalized the obtained parameters by the fraction of the image area occupied by the total are of the cells. For any given cell configuration on an image we calculated the corresponding Bar1 activity parameters. The volume of the containing medium was calculated by the image area multiplied with the height of the liquid film. This height h is given by the surface tension γ of the medium (aqueous solution), the gravitational acceleration g and the liquid density ρ via

$$h = 2\sqrt{\frac{\gamma}{g\rho}}. \tag{30}$$

This finally yielded the Bar1 activity with and without stimulation, which was plugged into the reaction-diffusion equations (also see Figure S5). The fitted Bar1 activity indicated a pheromone-dependent induction of about 3-fold which was in good agreement with previous measurements (3.4 ± 1.1 [8]). The approximated Bar1 activity in a typical liquid film ($h \approx 170\mu m$) where the bottom is covered completely with cells was derived as 0.24 s^{-1} without $\boldsymbol{\alpha}$-factor induction and could rise to 0.84 s^{-1} when induced by $\boldsymbol{\alpha}$-factor. This is a significant degradation activity, however, it is strongly reduced on lower cell densities. For a cell density of 1 Mio. cells per ml the Bar1 activity is 0.00053 s^{-1} to 0.002 s^{-1}. Thus, the Bar1 activity is strongly coupled to the cell abundance in the medium and can reach a substantial value in high density cultures.

Estimating the average α-factor secretion rate

The observed local $\boldsymbol{\alpha}$-factor boundary value α_i for the $MAT\mathbf{a}$ cell i was fitted to the simulated $\boldsymbol{\alpha}$-factor concentration I_{α_i} (compare definition equation (8)) on the $MAT\mathbf{a}$ cell wall. Hence, to estimate the surface flux J_α we solve the optimization problem:

$$\text{Find } J_\alpha \text{ such that for } \boldsymbol{\alpha} = \{\alpha_i\} : \sum_{i=1}^{N_a} (I_{\alpha_i} - \alpha_i)^2 \to \min. \tag{31}$$

Here, we computed α_i by solving the stationary equations (16) and (17), where the computational domain is based on the corresponding microscopic image as described in the section "Construction of the computational

domain for simulations". The BFGS method was used with a numeric approximation of the derivatives. We also implemented the Nelder-Mead method which does not require derivatives but uses more function evaluations in order to test the robustness of the results. Both optimizers led to the same results. For optimization problems with about 10 to 20 cells this resulted in a computation time of less than 10 minutes per image and CPU (3 GHz) to reach a minimum for meshes with more than 20.000 triangles. We fitted the secretion rate of $\boldsymbol{\alpha}$-factor to 10 images obtained on 2 different days on a total of 52 $MAT\boldsymbol{\alpha}$ and 90 $MAT\mathbf{a}$ cells. Significance was assessed for all fits using the F-test for a zero-intercept-one-parameter model where 9 of the fits were found to be significant. The total secretion per cell could be calculated by multiplying the resulting flux (in $nM \cdot \mu m^2 s^{-1}$) by the average cell circumference calculated from the mean cell radius from the CASY counter experiments (yielding a unit of $nM \cdot \mu m^3 s^{-1} = 10^{-15} nmol/s$, see Figure 7 main text). The resulting average secretion rate was 865 molecules/s per $MAT\boldsymbol{\alpha}$ cell. The validity of the model and obtained parameters was tested by reliably predicting the fluorescence values from an independent image not used in the optimization (F-test p-value $< 2.2 \cdot 10^{-6}$, 67 $MAT\boldsymbol{\alpha}$ cells and 78 $MAT\mathbf{a}$ cells). The mean secretion rate $\hat{J}_\alpha$ obtained form the 9 significant fits was used here $(101.589 \cdot 10^{-15} nmol \cdot \mu m^{-1} s^{-1})$.

It is noteworthy that the presented methodology does not require a special functional form of the calibration (or response curve). Even though many cellular signaling systems show a Hill curve in their input-output relation, this is not a requirement. As long as the input-output relation is strictly monotonous all our methods can still be used with the only difference that the fit to a Hill curve may be substituted by some non-parametric curve such as a normal or smoothed spline or a sliding mean approach. This "black box" treatment of input-output relation allows to apply the methodology to systems in which the actual mechanisms of intracellular signaling are still unclear.

Analysis of α-factor distributions

In order to analyze key properties of the influence of Bar1 we analyzed the $\boldsymbol{\alpha}$-factor distribution independent of the exact culture composition and under varying population densities. We calculated the $\boldsymbol{\alpha}$-factor distribution for different randomly generated cell populations in an area of $150\mu m \times 150\mu m$. The populations were composed of half $MAT\mathbf{a}$ and $MAT\boldsymbol{\alpha}$ cells and cells where assumed to have a circular shape with radii sampled from the normal distribution extracted from a statistic of 730 individual cells. Since we needed particularly the radii in the xy-plane, those were taken from the zero samples of the calibration curves ($\mu = 1.98\mu m$, $\sigma_r = 0.25\mu m$). This was done for populations sizes of 2 up to 700 cells, roughly corresponding to a range of 20 up to 10,000 cells per mm^2. For each population density we calculated 16 repetitions with random cell configurations.

In a first step we calculated the maximum information content of the observed $\boldsymbol{\alpha}$-factor distribution. The rationale here is that the cells try to obtain information about their environment and, therefore, it is interesting to quantify the maximum amount of information the cells can perceive. The maximum information any distribution can carry is given by the entropy of this distribution. We started by taking the overall $\boldsymbol{\alpha}$-factor distribution from all pixels of the simulated image ($N = 512 \times 512$ points). The observed $\boldsymbol{\alpha}$-factor concentrations were subdivided into N_{bins} bins and the number of bins chosen applying Sturges' law

$$N_{bins} = \lceil \log_2 N + 1 \rceil. \tag{32}$$

This was used to obtain a discrete distribution from which the bin probabilities p_i ($i = 1, \dots, N_{bins}$) were approximated using a shrinkage estimator [9]. The entropy H, and thus the maximum information content I_{max}, was finally computed as

$$I_{max} = H = -\sum_{i=1}^{N_{bins}} p_i \ln p_i. \tag{33}$$

For each population density the entropy was averaged over the 16 replicates.

In a second step we calculated how well the direction of the $\boldsymbol{\alpha}$-factor signal could be perceived by the individual $MAT\mathbf{a}$ cells in the given setting. We calculated the relative difference of the points at the cell surface exposed to the maximum $\boldsymbol{\alpha}$-factor concentration (the front) and the points exposed to the minimum $\boldsymbol{\alpha}$-factor concentration (the back). Thus, the relative front/back difference d_i for the $MAT\mathbf{a}$ cell i is given by

$$d_i = \frac{\max\limits_{\mathbf{x} \in \Gamma_{a_i}} \alpha(\mathbf{x}, t) - \min\limits_{\mathbf{x} \in \Gamma_{a_i}} \alpha(\mathbf{x}, t)}{\min\limits_{\mathbf{x} \in \Gamma_{a_i}} \alpha(\mathbf{x}, t)} \tag{34}$$

The relative differences were finally averaged over all $MAT\mathbf{a}$ cells and over all 16 replicates for a given population density.

Finally, we also tracked the average $\boldsymbol{\alpha}$-factor concentration over all replicates in each population density in order to see how much the overall $\boldsymbol{\alpha}$-factor concentration changes with varying population densities considering that $MAT\mathbf{a}$ cells only respond to a small range of $\boldsymbol{\alpha}$-factor concentrations [6, 7]. We performed experiments with a population with equally mixed $MAT\mathbf{a}$ wt and $MAT\boldsymbol{\alpha}$ for and for six different initial numbers of cells (compare Table S2). The cells were sedimented in a circular dish with a diameter of $3.6cm$ in a solution of $2ml$. Assuming that the cells are placed on quadratic lattices on the ground of the dish we can caluclate the average distance between the cells. For this calcluation we also take the cell diameter into account, which is assumed to be $5\mu m$.

Numerical methods

The stationary model equations (16)-(17) as well as the dynamic model equations (13)-(14) were numerically solved by a discontinuous Galerkin finite element method (dG-FEM). This method can be used to approximate solutions of various types of partial differential equations. A general overview can be found in [10, 11]. As in classical finite element methods (FEM) the computational domain is divided into small grid elements, usually triangles (2D) or tetrahedra (3D). The grids can be unstructured, i.e. the grid elements can vary in their form, which allows the representation of complex geometries of computational domains. In the dg-FEM discretization this is done by discontinuous functions, which are piecewise linear or polynomials of higher order. The *non-symmetric interior penalty Galerkin* (NIPG) method is used here [12, 13]. Since dG-FEM methods are locally mass conservative, they are especially suitable for diffusion problems [11]. For the time discretization of equations (13)-(14) we applied a diagonally implicit Runge-Kutta method of order three. The spatial and temporal discretization yields a convergence rate of $\mathcal{O}(h^p) + \mathcal{O}(\tau^3)$ in the $\ell^\infty(L^2)$ - as well as in the $\ell^2(H^1)$-norm, where τ is the length of the longest time step, h is the mesh size and p is the polynomial degree.

Implementation

In our implementation we used the Distributed and Unified Numerics Environment (DUNE) [14]. In particular, we used the PDELab environment, where several FEM methods and the dG-FEM discretization are implemented. We applied the grid implementation UGGrid [15] to create meshes based on Gmsh [4] files. To solve the resulting linear equation systems we used SuperLU [16].

References

[1] Gordon A, Colman-Lerner A, Chin TE, Benjamin KR, Yu RC, et al. (2007) Single-cell quantification of molecules and rates using open-source microscope-based cytometry. Nat Methods 4: 175–181.

[2] Chernomoretz A, Bush A, Yu R, Gordon A, Colman-Lerner A (2008) Using Cell-ID 1.4 with R for microscope-based cytometry. Curr Protoc Mol Biol Chapter 14: Unit 14.18.

[3] R Development Core Team (2012) R: A Language and Environment for Statistical Computing. R Foundation for Statistical Computing, Vienna, Austria. URL `http://www.r-project.org`. ISBN 3-900051-07-0.

[4] Geuzaine C, Remacle JF (2008) Gmsh: a three-dimensional finite element mesh generator with built-in pre- and post-processing facilities. International Journal for Numerical Methods in Engineering .

[5] Fischer H, Polikarpov I, Craievich A (2004) Average protein density is a molecular-weight-dependent function. Protein Science 13: 2825–2828.

[6] Yu RC, Pesce CG, Colman-Lerner A, Lok L, Pincus D, et al. (2008) Negative feedback that improves information transmission in yeast signalling. Nature 456: 755–761.

[7] Malleshaiah M, Shahrezaei V, Swain P, Michnick S (2010) The scaffold protein Ste5 directly controls a switch-like mating decision in yeast. Nature 465: 101–105.

[8] Manney TR (1983) Expression of the BAR1 gene in Saccharomyces cerevisiae: induction by the alpha mating pheromone of an activity associated with a secreted protein. J Bacteriol 155: 291–301.

[9] Hausser J, Strimmer K (2008) Entropy inference and the James-Stein estimator, with application to non-linear gene association networks. Journal of Machine Learning Research 10:: 1469–1484.

[10] Arnold D, Brezzi F, Cockburn B, Marini L (2002) Unified analysis of discontinuous Galerkin methods for elliptic problems. SIAM journal on numerical analysis : 1749–1779.

[11] Rivière B (2008) Discontinuous Galerkin Methods for Solving Elliptic an Parabolic Equations - Theory and Implementation. SIAM, Frontiers in Applied Mathematics.

[12] Dolešji V, Feistauer M, Kučera V, Sobotíková V (2008) An optimal $L^{\infty}(L^2)$-error estimate of the discontinuous Galerkin method for a nonlinear nonstationary convection-diffusion problems. IMA J Numer Anal 3: 496–521.

[13] Béatrice Rivière MFW, Girault V (1999). Improved Energy Estimates for Interior Penalty, Constrained and Discontinuous Galerkin Methods for Elliptical Problems.

[14] Bastian P, Blatt M, Dedner A, Engwer C, Klöfkorn R, et al. (2008) A Generic Grid Interface for Parallel and Adaptive Scientific Computing. Part I: Abstract Framework. Computing 82: 103–119.

[15] Bastian P, Birken K, Johannsen K, Lang S, Neuß N, et al. (1997) UG–a flexible software toolbox for solving partial differential equations. Computing and Visualization in Science 1: 27–40.

[16] Li X (2005) An overview of SuperLU: Algorithms, implementation, and user interface. ACM Transactions on Mathematical Software (TOMS) 31: 302–325.

[17] Rappaport N, Barkai N (2012) Disentangling signaling gradients generated by equivalent sources. J Biol Phys 38: 267–78.

[18] Rogers DW, McConnell E, Greig D (2012) Molecular quantification of Saccharomyces cerevisiae alpha-pheromone secretion. FEMS Yeast Res 12: 668–74.

[19] Jin M, Errede B, Behar M, Mather W, Nayak S, et al. (2011) Yeast dynamically modify their environment to achieve better mating efficiency. Sci Signal 4: ra54.

[20] Andrews SS, Addy NJ, Brent R, Arkin AP (2010) Detailed Simulations of Cell Biology with Smoldyn 2.1. PLoS Computational Biology 6.

Figures S1 to S8

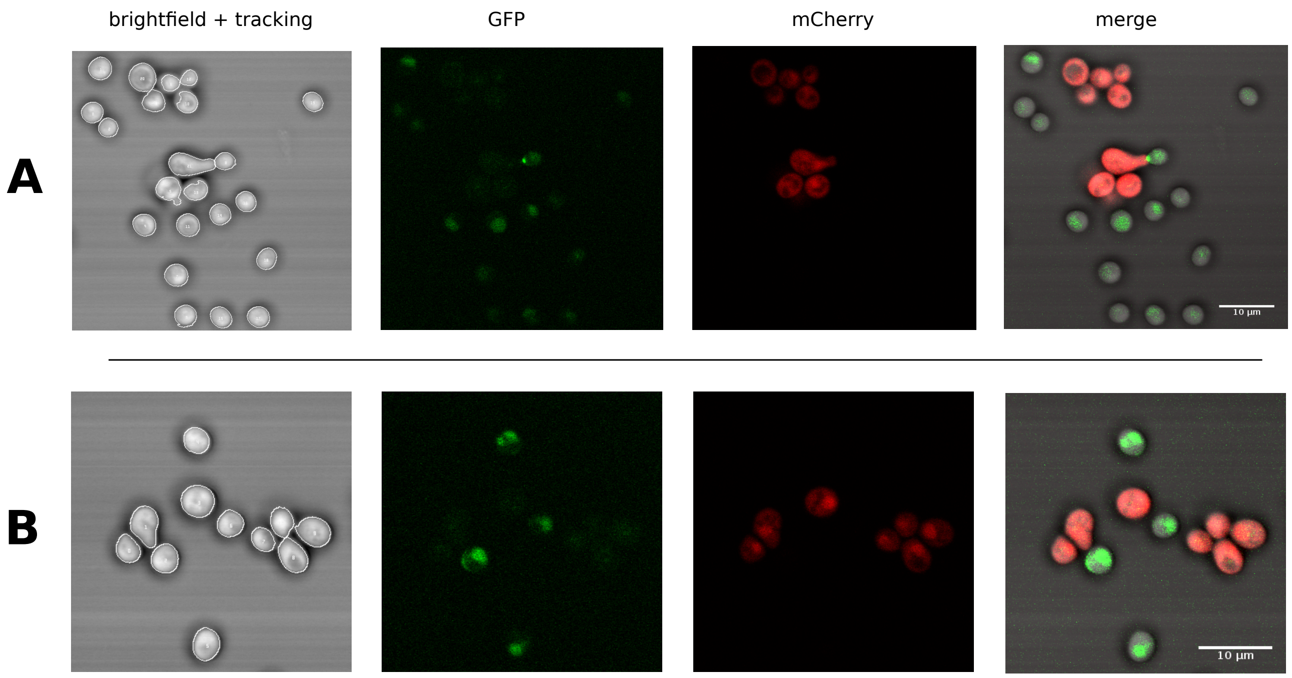

Figure S1. Raw confocal images and merging. Confocal images as taken from the microscope and merged in Figure 1 and 3 of the main text. Individual channels were obtained as described in this Supplementary Text. Individual cells were identified directly from brightfield images. The GFP channel was used for Fus1-GFP or Rpl9A-GFP fluorescence and the red channel for mCherry fluorescence under the *TDH3* promoter.

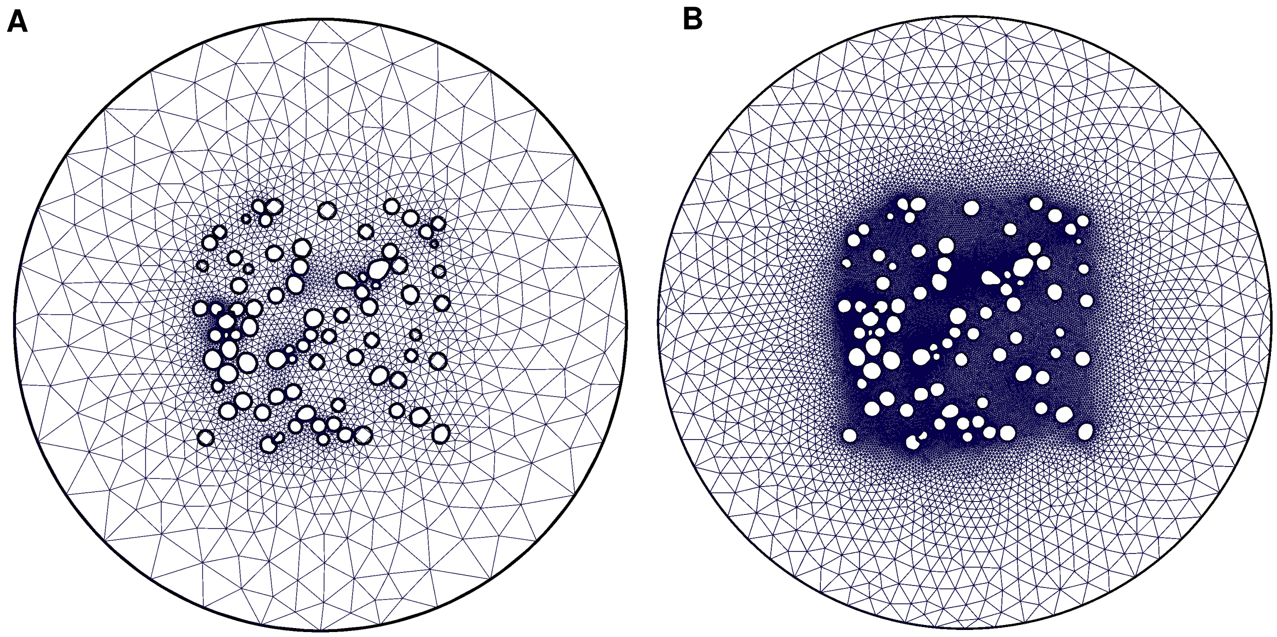

Figure S2. Examples of automatically generated meshes. Meshes as automatically generated from CellID output. Depending on the parameters one can extract meshes with varying coarseness.

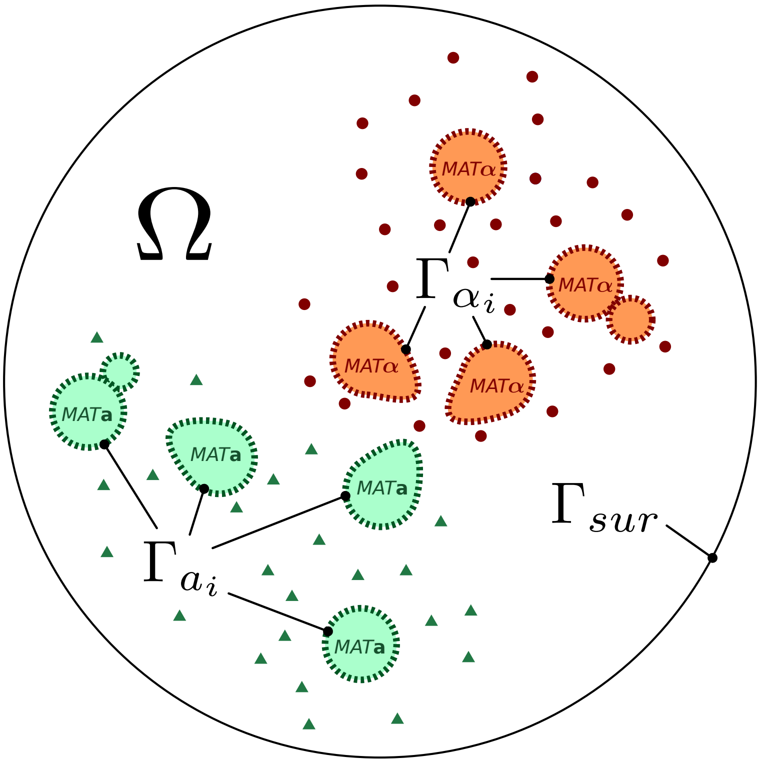

Figure S3. Schematic representation of the composition of the computational domain Ω. The interior of the cells is excluded from Ω. The boundaries are denoted by Γ_{α_i}, Γ_{a_i} and Γ_{sur}. Green triangles and red dots indicate Bar1 and **α**-factor, respectively.

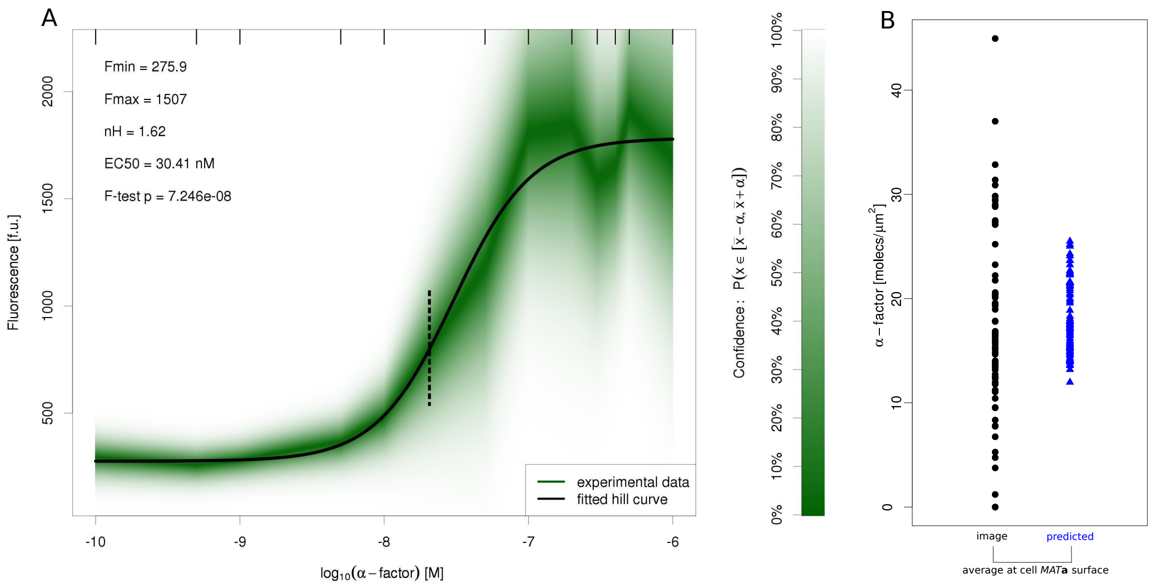

Figure S4. The Fus1-GFP calibration curve and predicted GFP levels. (A) The calibration used to associate the Fus1-GFP fluorescence with local $\boldsymbol{\alpha}$-factor concentrations. The green band shows the distribution of the measured fluorescence values. The color intensity indicates the confidence, thus the probability to observe a Fus1-GFP fluorescence within the region of the band with a distinct intensity (2965 MAT**a** cells in total). The black rugs on the upper axis indicate the concentrations at which measurements have been taken. One can observe that the relation between local $\boldsymbol{\alpha}$-factor concentration and Fus1-GFP fluorescence is sufficiently unique for small $\boldsymbol{\alpha}$-factor concentrations (below 100 nM). Relevant $\boldsymbol{\alpha}$-factor concentrations observed in the experiments with wild type mating mixes never exceeded 20 nM (dashed line). (B) The observed and predicted average $\boldsymbol{\alpha}$-factor concentrations on the surface of the MAT**a** cells in the verification image. The prediction seems to reduce the variance observed in the measurements. The fit was significant under a one-parameter F-test ($p < 2.2e-6$).

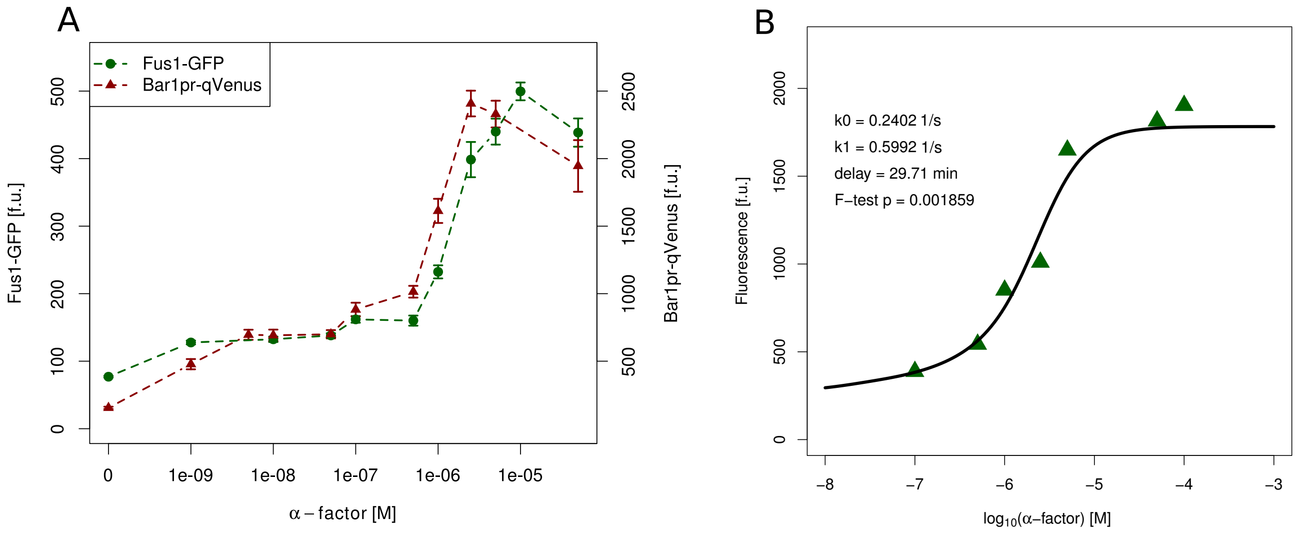

Figure S5. Fit of the extracellular Bar1 activity. (A) Comparison of the Fus1-GFP expression to the Bar1pr-qVenus expression measured by fluorescence. Error bars denote standard deviation (643 cells in total for Fus1-GFP and 578 cells for Bar1pr-qVenus). (B) Results of approximating the Bar1 activity kinetics. The green triangles indicate the mean values of the measured fluorescences (2.458 MATa cells in total). Parameters were obtained by maximizing the log-likelihood under the data by using the entire distributions of fluorescence values at each measured concentration of added $\boldsymbol{\alpha}$-factor. The Bar1 activities are normalized by the cell number per volume. Experiments in (A) and (B) used different GFP acquisition parameters.

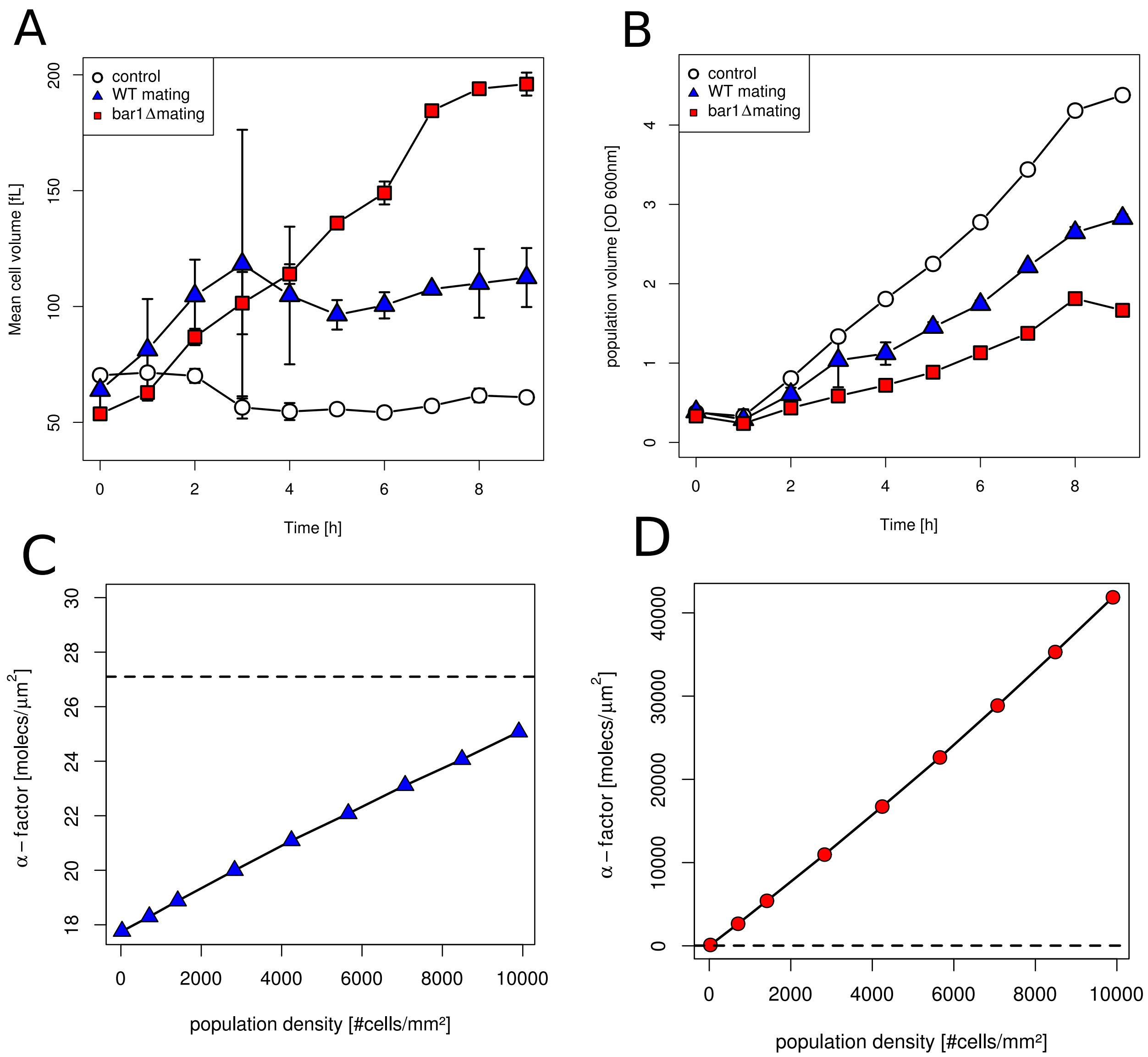

Figure S6. Additional experimental and computational data. (A) Volumes of the cells in different conditions. Cells in the $MAT\mathbf{a}$ $bar1\Delta/MAT\boldsymbol{\alpha}$ mating mix background show higher average volumes, which increase with time, whereas wild type cultures increase their volume to that of the size of diploids and maintain this over time (n=6). (B) Growth curves for mating cultures and haploids alone (control). The higher growth rate of the wild type compared to the mating $bar1\Delta$ culture is also observed here (two biological replicates). However, one can also observe that OD is a poor approximation for the number of cells in the population since the increase in OD of the $bar1\Delta$ culture is entirely due to the larger cell volumes (OD and individual cell volumes increase about 4-fold). (C) Average $\boldsymbol{\alpha}$-factor concentration on the simulated in vitro cell populations in the wild type background. (D) Average $\boldsymbol{\alpha}$-factor concentration in the simulated populations in the $bar1\Delta$ background. The dashed lines indicate $10nM$, which was the minimum concentration in the calibration curve were shmoo formation was observed.

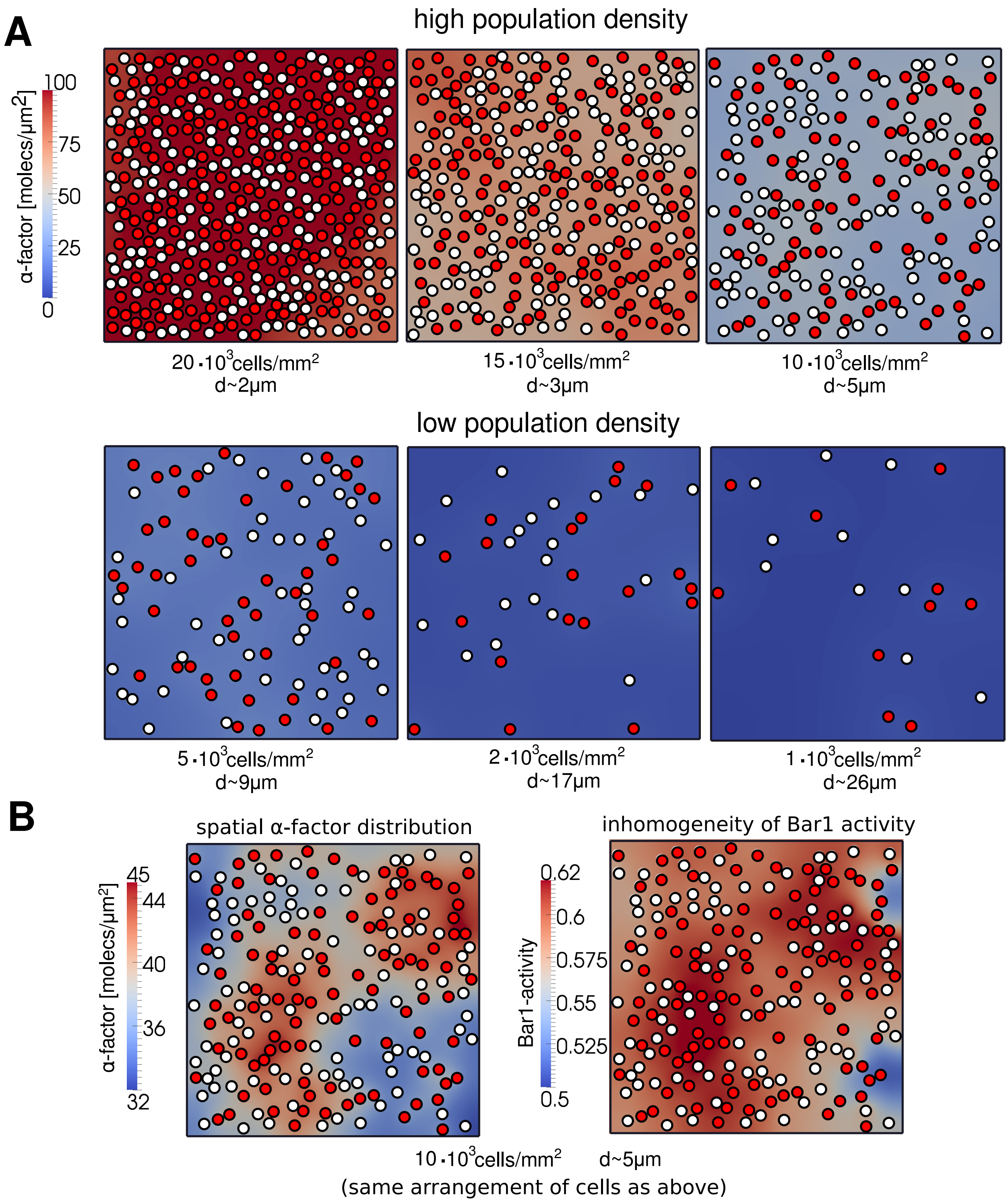

Figure S7. Density dependence of α-factor level and gradient formation. (A) Computational experiments have been performed with random cell arrangements for mixed populations (50% $MAT\boldsymbol{\alpha}$, 50% $MAT\mathbf{a}$). The densities of these populations correspond to the experiments shown in Figure 5. (B) The simulation from above [top row, on the right] for a density of $10 \cdot 10^6 cells/ml$ is plotted again using another color scale to make $\boldsymbol{\alpha}$-factor gradients visible [left]. For the same arrangement the inhomogeneous Bar1 activity is depicted [right].

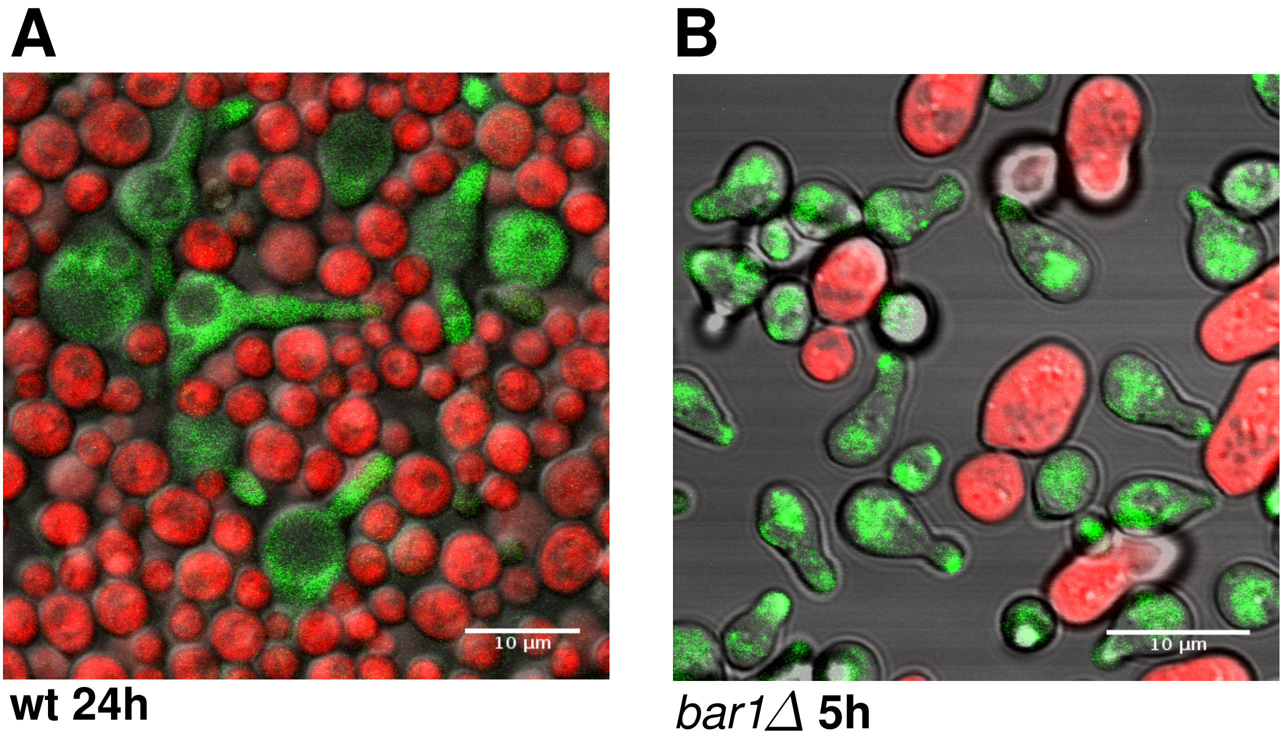

Figure S8. Phenotypes arising from long incubation. (A) Wild type *MAT***a** Fus1-GFP cells were mixed with high fractions of *MAT*$\boldsymbol{\alpha}$ mCherry and incubated overnight while rapidly shaken in order to prevent conjugation. (B) *MAT***a** Fus1-GFP *bar*1Δ cells together with *MAT*$\boldsymbol{\alpha}$ mCherry incubated without shaking, thus with $\boldsymbol{\alpha}$-factor gradients (also compare Figure 7 in the main text). The behavior already starts after 5h were the entire culture is in arrest. Comparing both images evidences that shaking of the wild type cultures, and thus disrupting the $\boldsymbol{\alpha}$-factor distribution, has a similar effect as a deletion of Bar1 and results in elongated cells with high Fus1-GFP expression.

Tables S1 and S2

Quantity	Our	Others
Secretion of α-factor per cell	$865\frac{molec}{s}$	$10\frac{molec}{s}$ [17]; $550-1300\frac{molec}{s}$ [18]; $3000\frac{molec}{s}$ [19]; $1230-4000\frac{molec}{s}$ [20]
Diffusion coefficient for α-factor	$361\frac{\mu m^2}{s}$	$300\frac{\mu m^2}{s}$ [17]; $125\frac{\mu m^2}{s}$ [19]; $132\frac{\mu m^2}{s}$ [20]
Diffusion coefficient for Bar1	$104\frac{\mu m^2}{s}$	$30\frac{\mu m^2}{s}$ [17]; $6.25\frac{\mu m^2}{s}$ [19]; $27\frac{\mu m^2}{s}$ [20]
Bar1 induction by α-factor stimulation	yes, fitted Hill-curve	no [17], [20], [19]
Bar1 localization	free diffusion in the media	free diffusion in the media [19], [20]; cell-wall-bound [17]
Bar1 activity	$0.24s^{-1}-0.84s^{-1}$	$1/60s^{-1}$ [17]
Endocytosis of α-factor by MAT**a** cells	no	no [19]; buffered [20]; MAT**a** cells are perfect absorbers [17]

Table S1. A comparison of parameters and assumptions in recent works. References correspond to reference list in Supplementary Text S1.

number of cells	cell density	OD_{600}	average distance	cells per area
$1\cdot10^6$	$0.5\cdot10^6\ cells/ml$	0.017	$26.6\mu m$	$1\cdot10^3\ cells/mm^2$
$2\cdot10^6$	$1\cdot10^6\ cells/ml$	0.033	$17.4\mu m$	$2\cdot10^3\ cells/mm^2$
$5\cdot10^6$	$2.5\cdot10^6\ cells/ml$	0.083	$9.1\mu m$	$5\cdot10^3\ cells/mm^2$
$10\cdot10^6$	$5\cdot10^6\ cells/ml$	0.16	$5.0\mu m$	$10\cdot10^3\ cells/mm^2$
$15\cdot10^6$	$7.5\cdot10^6\ cells/ml$	0.25	$2.7\mu m$	$15\cdot10^3\ cells/mm^2$
$20\cdot10^6$	$10\cdot10^6\ cells/ml$	0.33	$2.1\mu m$	$20\cdot10^3\ cells/mm^2$

Table S2. Initial cell numbers as used in the density experiments (Figure 5 and Supplementary Figure S7) and average distances between cells.
