# Supplementary material for: Yeast Mating and Image-Based Quantification of Spatial Pattern Formation
Source: PLoS Comput Biol. 2014 Jun 26;10(6):e1003690. doi: 10.1371/journal.pcbi.1003690 (PMC4072512; doi:10.1371/journal.pcbi.1003690)
Supplement: Text S2 — Comparison between 2D and 3D model formulations. Computational quantification of the relation between the 2D and 3D model for several arrangements of MAT α and MAT a cells are shown in Figures S9 to S11. The transformation of important quantities is listed in Table S3 and computational results are shown in Table S4. (PDF) [file pcbi.1003690.s003.pdf]

Supporting Text S2 for Yeast mating and image-based quantification of spatial pattern formation

Christian Diener[1*], Gabriele Schreiber[1*], Wolfgang Giese[1*], Gabriel del Rio[2], Andreas Schröder[3], Edda Klipp [1*]

[1*] Theoretische Biophysik, Humboldt-Universität zu Berlin,
Invalidenstr. 42, 10115 Berlin, Germany

[2] Institutio de Fisilogía Celular, Universidad Nacional Autónoma de México
S/N Cuidad Universitaria, 04510 México D.F, México

[3] Department of Mathematics, University of Salzburg,
Hellbrunnerstr. 34, 5020 Salzburg, Austria

*Correspondence to: edda.klipp@biologie.hu-berlin.de

Contents

Experimental setup 2

Relation between 2D and 3D model equations 2

Simulation results 3

Tables S3 and S4 7

Experimental setup

In the experiments the cells sedimented on the ground and were covered by a fluid film with a height of approximately $170\mu m$ (compare Figure S9). The height of the fluid film is large in comparison to the cell radius, which is on average $2\mu m$. As stated in the main text the pheromone concentration was integrated over the z-Axis, i.e. over the whole fluid film from $z = 0$ to $z = 170\mu m$. This yields a simplification of our problem from three to two space dimensions. In the next section we will describe in detail how we established a connection between the 2D and the full 3D model equations. In addition, the assunptions we made are verified by a comparison of a number of 2D and 3D simulations.

Relation between 2D and 3D model equations

The model equations in Supplementary Text S1 (13) - (14) describe a reaction diffusion process in three space dimensions. The concentration of $\boldsymbol{\alpha}$-factor as well as the Bar1-activity is integrated along the z-Axis over the whole fluid film of height $h = 170\mu m$. This yields functions in two space dimensions:

$$\alpha^{2D}(x,y,t) = \int_0^h \alpha(x,y,z,t)dz \text{ and } B^{2D}(x,y,t) = \int_0^h B(x,y,z,t)dz.$$

Since Bar1 is not degraded, the Bar1-activity is assumed to reach a steady state, which is nearly homogeneous along the z-Axis. Therefore, the approximation $B^{2D}(x,y,t) \approx h \cdot B(x,y,0,t)$ is used. For the nonlinear reaction term on the right hand side of equation Supplementary Text S1 (13) we get:

$$\begin{aligned}\int_0^h B(x,y,z,t)\cdot\alpha(x,y,z,t)dz &\approx \int_0^h B(x,y,0,t)\cdot\alpha(x,y,z,t)dz \\ &= B(x,y,0,t)\cdot\int_0^h \alpha(x,y,z,t)dz \\ &= \tfrac{1}{h}B^{2D}(x,y,t)\cdot\alpha^{2D}(x,y,t).\end{aligned}$$

This yields a system in two dimensions:

$$\begin{aligned}\frac{\partial\alpha^{2D}}{\partial t}(x,y,t) &= D_\alpha\Delta\alpha^{2D}(x,y,t) - \tfrac{1}{h}B^{2D}(x,y,t)\cdot\alpha^{2D}(x,y,t) \\ \frac{\partial B^{2D}}{\partial t}(x,y,t) &= D_B\Delta B^{2D}(x,y,t).\end{aligned}$$

The boundary conditions of α^{2D} are written as:

$$-D_\alpha\nabla\alpha^{2D}(x,y,t)\cdot\boldsymbol{n} = \begin{cases} J_\alpha^{2D} & \text{on } MAT\alpha\text{-cells,} \\ 0 & \text{otherwise .}\end{cases}$$

and for B^{2D} we have:

$$\begin{aligned}B^{2D}(x,y,t) &= C^{2D}(\alpha(t-\tau)) \text{ on the } MAT\mathbf{a}\text{-cells or} \\ -D_B\nabla B^{2D}(x,y,z,t)\cdot\boldsymbol{n} &= 0 \text{ otherwise.}\end{aligned}$$

It remains to relate the Flux J_α^{2D} and the Bar1 activity rate $C^{2D}(\alpha(t-\tau))$ in a consistent way with the corresponding three dimensional expressions. Due to mass conservation it holds:

$$\int_{S_{\alpha_i}} J_\alpha^{2D} ds = \int_{H_{\alpha_i}} J_\alpha^{3D} dA,$$

i.e. the pheromone secretion of a cell in 2D is the same as in 3D. Here S_i denotes a circle of radius r_i for the 2D case and H_{α_i} denotes a hemisphere of radius r_i for the 3D case, which serve as an approximation for a cell

of radius r_i. Therefore, we get the relation $2\pi r_i J_\alpha^{2D} = 2\pi r_i^2 J_\alpha^{3D}$, e.g. $J_\alpha^{2D} = r_i \cdot J_\alpha^{3D}$. Since the Bar1-activity is integrated over h it holds:

$$\begin{aligned} C^{2D}(\alpha(t-\tau)) = & \quad h \cdot C^{3D}(\alpha(t-\tau)) \\ = & \quad h \cdot \tilde{k}_0 + h \cdot \tilde{k}_1 \frac{I_{\alpha_i}(t-\tau)^H}{I_{\alpha_i}(t-\tau)^H + (r_i \cdot EC_{50})^H} \text{ on } \Gamma_{a_i}, \quad i = 1, \ldots, N_a. \end{aligned}$$

Thus, we have $\tilde{k}_0^{2D} = h \cdot \tilde{k}_0$, $\tilde{k}_1^{2D} = h \cdot \tilde{k}_1$ and $EC_{50}^{2D} = r_i \cdot EC_{50}$.

These transformed model equations are used for the simulations in two space dimensions. All the relations between 2D and 3D variables as well as constants that were derived in this section can be found in Table S3.

Simulation results

Simulations in three space dimensions with arrangments of three, five and seven *in silico* cells with varying pheromone fluxes were performed in a fluid film of height $h = 170\mu m$ (see Figures S9 and S10). We compared the steady state of the 3D simulations with those of the corresponding 2D simulations. For this comparison, the values of the 3D simulation were integrated over the z-axis as stated in the previous section. The three dimensional finite element mesh that was used for the arrangement of five cells is shown in (Figure S10). The experiments were performed with the parameters given in Tables S3 and S4. We found that the integrated distribution of $\boldsymbol{\alpha}$-factor in the two models is essentially the same (compare Figure S11). Also, average $\boldsymbol{\alpha}$-factor concentrations on the surface of the $MAT\mathbf{a}$ cells deviated less than 5 % of the maximum $\boldsymbol{\alpha}$-factor concentration, as indicated by the results presented in Table S4 for varoius pheromone fluxes $J_\alpha = 50.0, 101.589$ and 200.0 $nM \cdot \mu m^2/s$. The reduction to a 2D simulation also strongly lowers the computational complexity of the model. In our simulations the decline in computation time between 3D and 2D was from several hours to less than a minute. This allows for the quick optimization for key parameters as employed in this work.

Figures S9 to S11

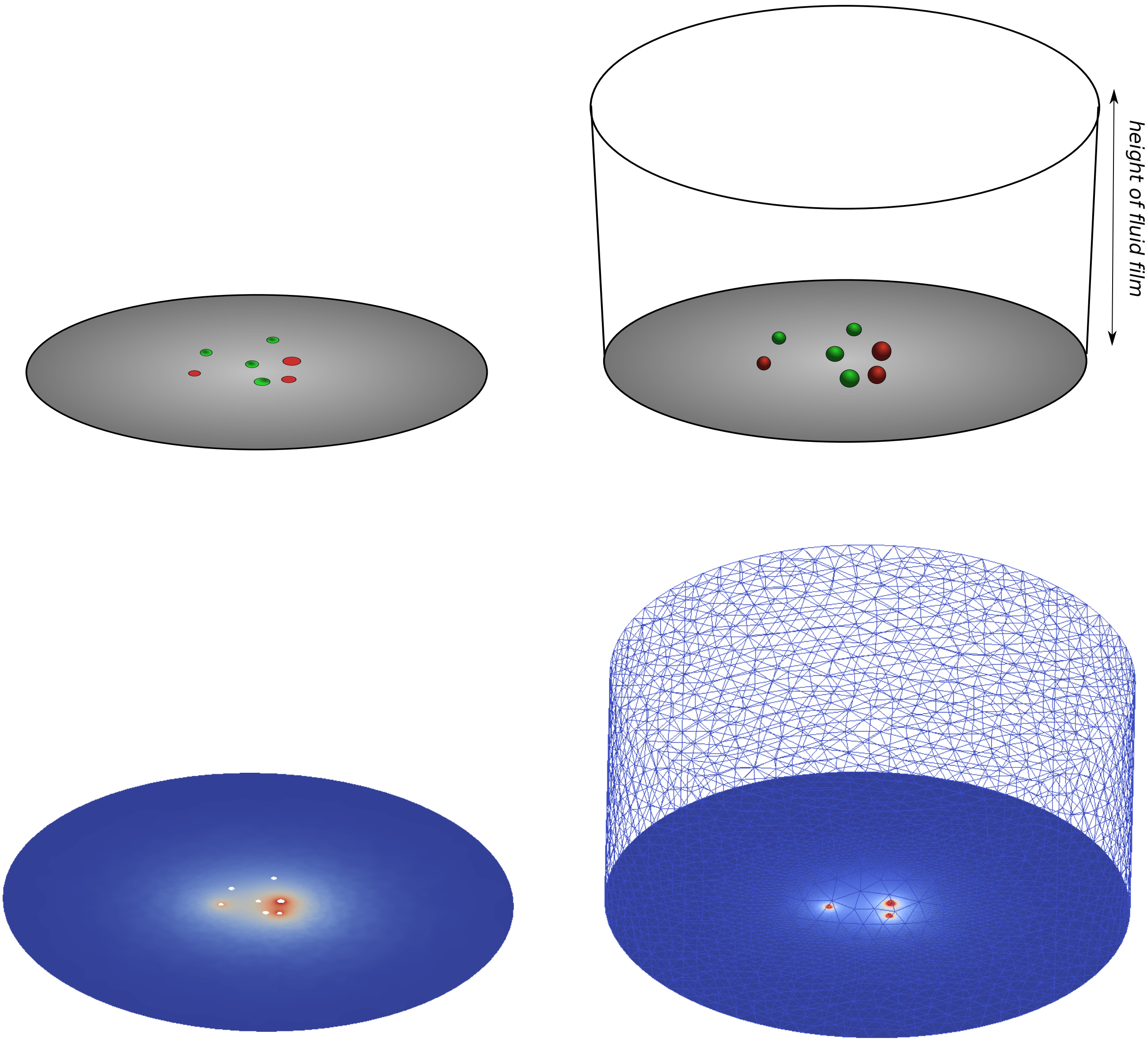

Figure S9. Comparison of 2D and 3D simulations. A simulation is shown for an arrangement of three $MAT\boldsymbol{\alpha}$ cells and four $MAT\mathbf{a}$ cells. The height of the 3D computational domain on the right corresponds to the height of the fluid film $h = 170\mu m$. The 2D simulation result on the left shows the pheromone distribution with the transformed 2D parameters.

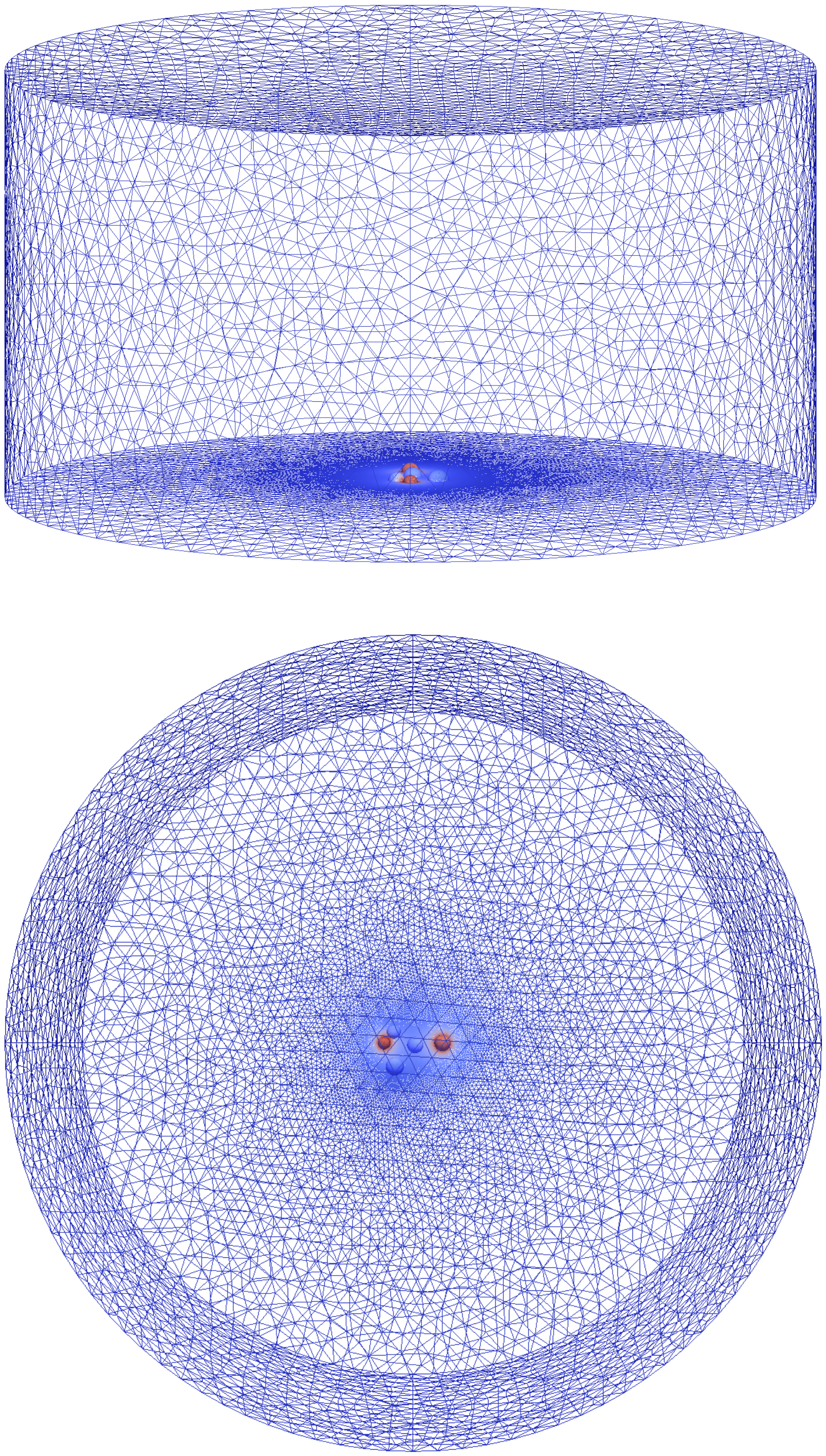

Figure S10. The finite element mesh corresponding to the 3D computational domain for five in silico cells – frontal view and view from top. The mesh consists of 207.012 mesh elements.

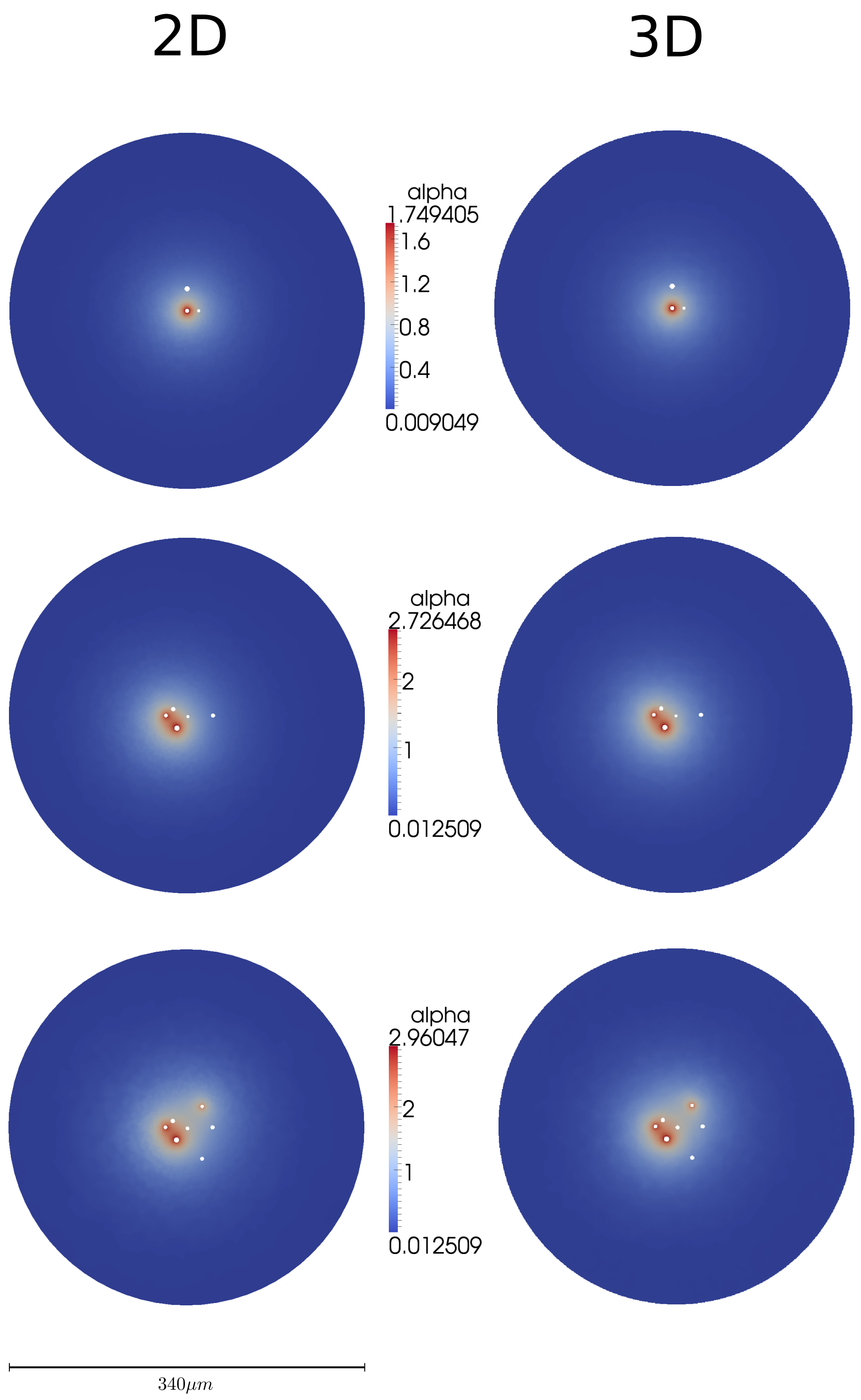

Figure S11. Comparison of the 2D simulations with the integrated values of corresponding 3D simulations. Shown is a simulation of of two $MAT\boldsymbol{\alpha}$ cells and three $MAT\mathbf{a}$ cells. The differences of the integrated values from the 3D simulation and the values obtained in the 2D simulation are shown in Supplementary Table S4.

Tables S3 and S4

	2D	3D
Pheromone concentration	$\alpha^{2D}(x,y,t) = \int_0^h \alpha(x,y,z,t)dz$, unit $[nM \cdot \mu m]$	$\alpha(x,y,z,t)$, unit $[nM]$
Bar1 concentration	$Bar1^{2D}(x,y,t) = \int_0^h Bar1^{2D}(x,y,z,t)dz$, unit $[nM \cdot \mu m]$	$Bar1(x,y,z,t)$, unit $[nM]$
Degradation rate	$k^{2D} = \frac{1}{h}k$, unit $\left[nM^{-1} \cdot \mu m^{-1} \cdot s^{-1}\right]$	k, unit $\left[nM^{-1} \cdot s^{-1}\right]$
Bar1 activity	$B^{2D}(x,y,t) = \int_0^h B^{2D}(x,y,z,t)dz$ $\approx h \cdot B(x,y,0,t)$, unit $\left[\mu m \cdot s^{-1}\right]$	$B(x,y,z,t) = k \cdot Bar1(x,y,z,t)$, unit $\left[s^{-1}\right]$
Rate constants for Bar1	$\tilde{k}_0^{2D} = h \cdot \tilde{k}_0 = 40.8\mu ms^{-1}$, $\tilde{k}_1^{2D} = h \cdot \tilde{k}_1 = 102.0\mu ms^{-1}$	$\tilde{k}_0 = 0.24s^{-1}$, $\tilde{k}_1 = 0.6s^{-1}$
Diffusion constants	$D_\alpha = 361.48\mu m^2 \cdot s^{-1}$, $D_B = 104.63\mu m^2 \cdot s^{-1}$	the same
pheromone fluxes	J_α, unit $\left[nM \cdot \mu m^2 \cdot s^{-1}\right]$	$J_\alpha^{3D} = \frac{1}{r_i}J_\alpha$, unit $\left[nM \cdot \mu m \cdot s^{-1}\right]$
Kinetic constant	$EC_{50}^{2D} = r_i \cdot EC_{50}$, unit $[nM \cdot \mu m]$	$EC_{50} = 30,41nM$, unit $[nM]$
Hill coefficient	H (without unit)	the same
Height of the fluid layer	$h = 170\mu m$	the same

Table S3. Relations between 2D and 3D variables and constants.

setup	integrated $\boldsymbol{\alpha}$-factor	2D value	3D value	rel. error (ϵ/α_{max})
#Cells = 3				
$J_\alpha = 50.0 \frac{nM \cdot \mu m^2}{s}$	maximum	0.861483 $nM \cdot \mu m$	0.861208$nM \cdot \mu m$	0.0319318%
	average at $MAT\mathbf{a}$ #1	0.394679 $nM \cdot \mu m$	0.417416 $nM \cdot \mu m$	2.64012%
	average at $MAT\mathbf{a}$ #2	0.238334 $nM \cdot \mu m$	0.239569 $nM \cdot \mu m$	0.143403%
$J_\alpha = 101.589 \frac{nM \cdot \mu m^2}{s}$	maximum	1.7494 $nM \cdot \mu m$	1.74901 $nM \cdot \mu m$	0.0222983%
	average at $MAT\mathbf{a}$ #1	0.801022 $nM \cdot \mu m$	0.847345 $nM \cdot \mu m$	2.64852%
	average at $MAT\mathbf{a}$ #2	0.48353 $nM \cdot \mu m$	0.486125 $nM \cdot \mu m$	0.148369%
$J_\alpha = 200.0 \frac{nM \cdot \mu m^2}{s}$	maximum	3.43877 $nM \cdot \mu m$	3.43887 $nM \cdot \mu m$	0.00290793%
	average at $MAT\mathbf{a}$ #1	1.57202 $nM \cdot \mu m$	1.6639 $nM \cdot \mu m$	2.6718%
	average at $MAT\mathbf{a}$ #2	0.947904 $nM \cdot \mu m$	0.953478 $nM \cdot \mu m$	0.162088%
#Cells = 5				
$J_\alpha = 50.0 \frac{nM \cdot \mu m^2}{s}$	maximum	1.34443 $nM \cdot \mu m$	1.29098 $nM \cdot \mu m$	4.14026%
	average at $MAT\mathbf{a}$ #1	0.740936 $nM \cdot \mu m$	0.734099 $nM \cdot \mu m$	0.529597%
	average at $MAT\mathbf{a}$ #2	0.238287 $nM \cdot \mu m$	0.233003 $nM \cdot \mu m$	0.409301%
	average at $MAT\mathbf{a}$ #3	0.609594 $nM \cdot \mu m$	0.605616 $nM \cdot \mu m$	0.308138%
$J_\alpha = 101.589 \frac{nM \cdot \mu m^2}{s}$	maximum	2.72647 $nM \cdot \mu m$	2.61901 $nM \cdot \mu m$	4.10307%
	average at $MAT\mathbf{a}$ #1	1.50064 $nM \cdot \mu m$	1.48775$nM \cdot \mu m$	0.49217%
	average at $MAT\mathbf{a}$ #2	0.481481 $nM \cdot \mu m$	0.470991 $nM \cdot \mu m$	0.400533%
	average at $MAT\mathbf{a}$ #3	1.23423 $nM \cdot \mu m$	1.22691 $nM \cdot \mu m$	0.279494%
$J_\alpha = 200.0 \frac{nM \cdot \mu m^2}{s}$	maximum	5.33946 $nM \cdot \mu m$	5.13384$nM \cdot \mu m$	4.10307%
	average at $MAT\mathbf{a}$ #1	2.92806 $nM \cdot \mu m$	2.90788$nM \cdot \mu m$	0.393078%
	average at $MAT\mathbf{a}$ #2	0.933276 $nM \cdot \mu m$	0.913787$nM \cdot \mu m$	0.379618%
	average at $MAT\mathbf{a}$ #3	2.40599 $nM \cdot \mu m$	2.39554$nM \cdot \mu m$	0.203551%
#Cells = 7				
$J_\alpha = 50.0 \frac{nM \cdot \mu m^2}{s}$	maximum	1.46102 $nM \cdot \mu m$	1.40047 $nM \cdot \mu m$	4.32354%
	average at $MAT\mathbf{a}$ #1	0.43321 $nM \cdot \mu m$	0.428717 $nM \cdot \mu m$	0.320820%
	average at $MAT\mathbf{a}$ #2	0.785789 $nM \cdot \mu m$	0.765185 $nM \cdot \mu m$	1.47122%
	average at $MAT\mathbf{a}$ #3	0.876589 $nM \cdot \mu m$	0.841532 $nM \cdot \mu m$	2.50323%
	average at $MAT\mathbf{a}$ #4	0.351402 $nM \cdot \mu m$	0.321063 $nM \cdot \mu m$	2.16634%
$J_\alpha = 101.589 \frac{nM \cdot \mu m^2}{s}$	maximum	2.96047 $nM \cdot \mu m$	2.83957 $nM \cdot \mu m$	4.25768%
	average at $MAT\mathbf{a}$ #1	0.874952 $nM \cdot \mu m$	0.866728 $nM \cdot \mu m$	0.289621%
	average at $MAT\mathbf{a}$ #2	1.58904 $nM \cdot \mu m$	1.54912 $nM \cdot \mu m$	1.49868%
	average at $MAT\mathbf{a}$ #3	1.77307 $nM \cdot \mu m$	1.70416 $nM \cdot \mu m$	2.42677%
	average at $MAT\mathbf{a}$ #4	0.709377 $nM \cdot \mu m$	0.648727 $nM \cdot \mu m$	2.13588%
$J_\alpha = 200.0 \frac{nM \cdot \mu m^2}{s}$	max. $\boldsymbol{\alpha}$-factor	5.78471 $nM \cdot \mu m$	5.55769 $nM \cdot \mu m$	4.08479%
	average at $MAT\mathbf{a}$ #1	1.69407 $nM \cdot \mu m$	1.68234 $nM \cdot \mu m$	0.211058%
	average at $MAT\mathbf{a}$ #2	3.08748 $nM \cdot \mu m$	3.01889 $nM \cdot \mu m$	1.23414%
	average at $MAT\mathbf{a}$ #3	3.44735 $nM \cdot \mu m$	3.32361 $nM \cdot \mu m$	2.22646%
	average at $MAT\mathbf{a}$ #4	1.37159 $nM \cdot \mu m$	1.25722 $nM \cdot \mu m$	2.05786%

Table S4. Comparison of crucial quantities in 2D and 3D simulations. Simulations were performed for setups of three, five, and seven in silico cells with varying pheromone secretion rates J_α.
